# Expanding super-resolution imaging versatility in organisms with multi-confocal image scanning microscopy

**DOI:** 10.1093/nsr/nwae303

**Published:** 2024-08-27

**Authors:** Wei Ren, Meiling Guan, Qianxi Liang, Meiqi Li, Boya Jin, Guangxing Duan, Liya Zhang, Xichuan Ge, Hong Xu, Yiwei Hou, Baoxiang Gao, Sodmergen, Peng Xi

**Affiliations:** Department of Biomedical Engineering, College of Future Technology, Peking University, Beijing 100871, China; National Biomedical Imaging Center, Peking University, Beijing 100871, China; Department of Biomedical Engineering, College of Future Technology, Peking University, Beijing 100871, China; National Biomedical Imaging Center, Peking University, Beijing 100871, China; Key Laboratory of Computational Optical Imaging Technology, Chinese Academy of Sciences, Beijing 100094, China; Department of Biomedical Engineering, College of Future Technology, Peking University, Beijing 100871, China; National Biomedical Imaging Center, Peking University, Beijing 100871, China; School of Life Sciences, Peking University, Beijing 100871, China; Department of Biomedical Engineering, College of Future Technology, Peking University, Beijing 100871, China; National Biomedical Imaging Center, Peking University, Beijing 100871, China; School of Life Sciences, Peking University, Beijing 100871, China; School of Life Sciences, Peking University, Beijing 100871, China; Key Laboratory of Analytical Science and Technology of Hebei Province, College of Chemistry and Material Science, Hebei University, Baoding 071002, China; Airy Technologies Co. Ltd., Beijing 100086, China; Department of Biomedical Engineering, College of Future Technology, Peking University, Beijing 100871, China; National Biomedical Imaging Center, Peking University, Beijing 100871, China; Key Laboratory of Analytical Science and Technology of Hebei Province, College of Chemistry and Material Science, Hebei University, Baoding 071002, China; School of Life Sciences, Peking University, Beijing 100871, China; Department of Biomedical Engineering, College of Future Technology, Peking University, Beijing 100871, China; National Biomedical Imaging Center, Peking University, Beijing 100871, China

**Keywords:** super-resolution imaging, confocal, image scanning microscopy

## Abstract

Resolving complex three-dimensional (3D) subcellular dynamics noninvasively in live tissues demands imaging tools that balance spatiotemporal resolution, field-of-view and phototoxicity. Image scanning microscopy (ISM), as an advancement of confocal laser scanning microscopy, provides a 2-fold 3D resolution enhancement. Nevertheless, the relatively low imaging speed has been the major obstacle for ISM to be further employed in *in vivo* imaging of biological tissues. Our proposed solution, multi-confocal image scanning microscopy (MC-ISM), aims to overcome the limitations of existing techniques in terms of spatiotemporal resolution balancing by optimizing pinhole diameter and pitch, eliminating out-of-focus signals, and introducing a frame reduction reconstruction algorithm. The imaging speed is increased by 16 times compared with multifocal structured illumination microscopy. We further propose a single-galvo scan, akin to the Archimedes spiral in spinning disk confocal systems, to ensure a high-speed and high-accuracy scan without the galvanometer's inertial motion. Benefitting from its high photon efficiency, MC-ISM allows continuous imaging of mitochondria dynamics in live cells for 1000 frames without apparent phototoxicity, reaching an imaging depth of 175 μm. Noteworthy, MC-ISM enables the observation of the inner membrane structure of living mitochondria in Arabidopsis hypocotyl for the first time, demonstrating its outstanding performance.

## INTRODUCTION

As biological research becomes more interested in model organisms such as early-stage embryos [1] and zebrafish [2], research limited to single-cell scale is insufficient to fully reveal the complex interactions and structures within multicellular tissues [3,4]. Therefore, deciphering the intricate three-dimensional subcellular dynamics in tissues with higher spatial complexity *in situ* is becoming increasingly crucial.

In the past two decades, the rapid development of super-resolution microscopy has provided new insights into many biological processes [5–7]. Super-resolution structured illumination microscopy (SIM) [8,9], which relies on high-frequency extraction from Moiré fringe, does not require special labeling methods, and is preferred for live-cell imaging due to its low-phototoxicity. However, SIM fails to reconstruct when imaging tissue samples with heavy scattering due to the submerged sinusoidal pattern. Stimulated emission depletion (STED) [10] microscopy, as another mainstream super-resolution imaging technique, provides higher resolution (30–50 nm) through the depletion of the doughnut-shaped STED beam. However, STED finds it difficult to maintain consistent resolution as the focus shifts from the sample surface to deep inside during depth imaging because of spherical aberration, scattering distortion, and attenuation [11–13]. The alternative single molecule localization microscopy tends to cause mis-localization owing to refractive index mismatch during depth imaging [14,15].

In contrast, confocal laser scanning microscopy has stood as the premier choice in depth imaging of scattered tissues due to its remarkable optical sectioning capabilities and versatility [16]. Image scanning microscopy (ISM) [17,18], as an advancement of confocal microscopy, provides outstanding optical sectioning capabilities while maintaining a 2-fold 3D resolution improvement by replacing the single-pixel detector with a detector array. Nevertheless, the relatively low imaging speed has been the major obstacle for the further employment of ISM in *in vivo* imaging of biological tissues. Various extended technologies of ISM have been developed based on ideas of optical reconstruction, or digital reconstruction of multi-focal parallel excitation [18–23]. Optical reconstruction-relying techniques, such as re-scan confocal microscopy [19], optical photon reassignment microscopy [20], instant SIM [21] and spinning disk confocal-optical photon reassignment [22] have effectively improved imaging speed, but the optical configuration is exceedingly complex. Also, the pixel reassignment requires descanning, rescanning, or passing through a microlens array for fluorescent signals, which results in substantial attenuation and a notable rise in phototoxicity. Moreover, even though complete optical reconstruction obviates the huge computational overhead, the potentials embedded in the redundant information from ISM remain underexplored.

Conversely, digital reconstruction technology exhibits lower phototoxicity which is attributed to its straightforward detection optical path. Digital reconstruction solutions, such as multifocal SIM (MSIM) [23] and confocal spinning-disk ISM [24], ingeniously leverage existing devices or modules to facilitate the seamless attainment of multi-focal data acquisition. MSIM uses a digital micromirror device (DMD) to generate a sparse multi-focus to implement the parallel excitation which achieves 1 Hz imaging speed within a 480-pixel field of view (FOV) [23]. However, first, the ratio of scanning step and pinhole size are constrained by the DMD hence an excessive number of original frames during the reconstruction process is required. Second, a redundant number of original frames is required to collect the overlapping information acquired by different pixels on the detector array [25], which also hinders imaging speed. The reconstruction algorithm is primarily based on pixel reassignment (PR), which can achieve a resolution enhancement of up to $\sqrt 2 $ times. Subsequent deconvolution can then be applied to achieve a final resolution improvement of 2 times. In addition to this approach, super-resolution images can also be directly reconstructed from the raw images through multi-image deconvolution [26], which necessitates an accurate estimation of the excitation pattern.

To solve the problem above, here, we propose multi-confocal image scanning microscopy (MC-ISM). Owing to the optimization of pinhole diameter and pitch, the elimination of out-of-focus signals facilitated by optical lock-in detection (OLID) [27] preprocessing, and the introduction of a multi-image deconvolution frame reduction reconstruction algorithm, imaging speed is increased by 16 times compared with multifocal structured illumination microscopy. Additionally, while maintaining the 2-fold enhancement in three-dimensional resolution, MC-ISM exhibits reduced phototoxicity and increased imaging depth. In the implementation, rejecting out-of-focus signals can be realized by sparse multi-focus illumination generated by the pinhole array combined with OLID. A single-galvo scan, akin to the Archimedes spiral in spinning disk confocal systems, was utilized to ensure a high-speed and high-accuracy scan by mitigating the inertial motion effect of the galvanometer. The fast iterative shrinkage-thresholding algorithm-group sparsity (FISTA-GS) [28,29] was proposed to utilize the redundant information to accomplish frame reduction reconstruction which accelerates the imaging speed. In addition, the implementation of a pinhole array with LED illumination can produce multi-focus with uniform intensity and constant pitch within a 100 μm FOV. Notably, MC-ISM enabled the observation of the inner membrane structure of living mitochondria in Arabidopsis hypocotyl for the first time, demonstrating its outstanding comprehensive performance.

## RESULTS

### Hardware implementation and scanning scheme

**Figure 1.**
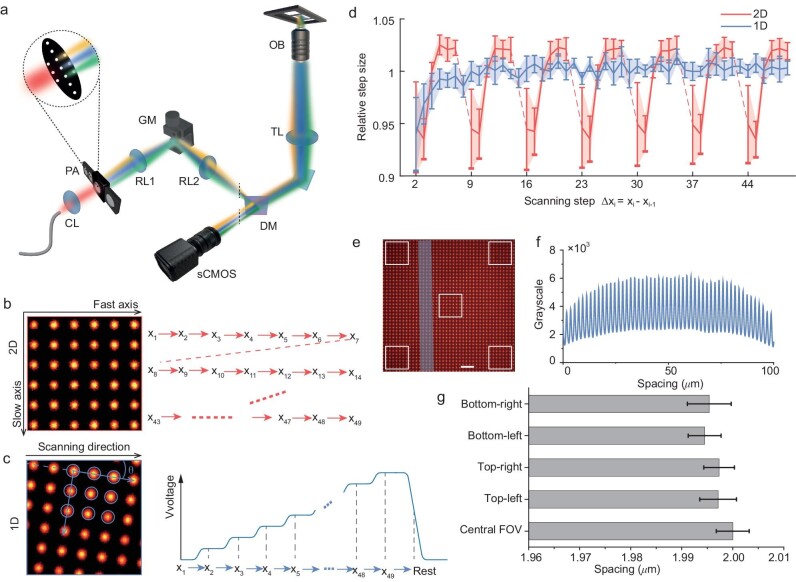
Hardware implementation and scanning scheme. (a) Optical setup of MC-ISM. CL: collimating lens; PA: pinhole array; RL1, RL2: relay lenses; GM: galvanometer mirror; DM: dichroic mirror; TL: tube lens; OB: objective lens. (b) 2D scanning point distribution and scanning method. (c) 1D scanning point distribution and scanning method. (d) 1D and 2D scanning step accuracy comparison. (e) Excitation points imaged onto an HBmito Crimson fluorescent dye sample. Scale bar: 10 μm. (f) Intensity profile measured along the long side of the blue rectangle area in Fig. 1e. (g) Select five white boxed areas (bottom-right, bottom-left, top-right, top-left, and central FOV) in Fig. 1e, measure the spacing between points, and draw a bar chart of spacing distribution (error bar: standard deviation (SD)).

Pinhole arrays fabricated by photolithography technology were utilized to generate multi-focus illumination (Fig. 1a) with much greater flexibility in optimizing the ratio between pinhole diameter and pitch, instead of using a microlens array which necessitate complex and expensive customized production with the possibility of introducing visible chromatic aberration in multicolor imaging [22]. Also, it is shown that pinhole arrays are compatible with single-mode lasers, multi-mode lasers and LEDs (Fig. S1). In this case, a high-power LED was selected as the illumination source since LEDs excel in eradicating speckle artifacts and offering expanded FOV illumination [30], and the relatively low transmission efficiency of pinhole arrays could be made up by the high power of the light source.

In previous studies [31,32], two-dimensional galvanometers were generally used to completely scan the entire FOV (Fig. 1b and Fig. S2). Here, a one-dimensional scanning manner by a single galvanometer is proposed. We generate an illumination pattern distributed at a tilted angle from the orthogonal coordinate system by rotating the pinhole array (Fig. 1c, Fig. S3, Note S1 and Movie S1) so that the spacing of its projection on the slow axis direction meets the sampling interval. Therefore, while the excitation pattern performs one-dimensional scanning along the fast axis, uniform illumination can also be achieved in the slow axis direction. Comparing the traditional two-dimensional scanning scheme employed by a pair of galvanometers, one-dimensional scanning has notable advantages: (1) one-dimensional scanning does not require alignment of total scan length in two dimensions with the pitch of pinhole array, which is beneficial to avoid undesirable artifacts; (2) the galvanometer steps forward continuously when scanning only in one dimension, while two-dimensional scanning needs retracing of galvanometer resulting in uneven scanning steps (Fig. 1d) and unnecessary heating; (3) only one galvanometer in one-dimensional scanning avoids the disturbance caused by vibration of the other galvanometer; (4) the single galvanometer is perfectly aligned with the entrance pupil of the objective to ensure maximum light throughput without vignetting. One-dimensional scanning by a single galvanometer not only simplifies the scanning process but also enhances image quality and system stability, ensuring the most accurate and artifact-free results across the FOV.

The optimal comprehensive performance of the system depends on critical factors such as the excitation pinhole diameter and scanning step. Simulation results indicate that smaller excitation pinholes can yield higher resolution, but at the expense of a proportional reduction in signal volume. Therefore, we selected a 40-μm-diameter pinhole array, equivalent to 0.5 Airy Unit (AU), to serve as the excitation pinhole (Fig. S4a–c). For pixel reassignment-based reconstruction, it is generally recommended to oversample the scanning step by a factor of two. Through simulations, we determined that denser sampling fails to enhance resolution but prolongs image acquisition time. Therefore, we adopted a scanning interval of 0.25 AU, which we deemed sufficient (Fig. S4d). An in-depth description is given in Note S2.

A uniform fluorescent plate (HBmito Crimson [33] fluorescent dye sample) was used to visualize the multifocal illumination (Fig. 1e), and a striped area was selected to evaluate the uniformity of 100 μm FOV (100 × objective). Leveraging LED as a light source has substantially enhanced the uniformity of the FOV (Fig. 1f). Five areas (bottom-right, bottom-left, top-right, top-left, and central FOV) within the FOV were selected where the distance (mean±SD) between adjacent points are calculated to evaluate distortion (Fig. 1g). The disparity in point spacing between the central and edge FOV does not exceed 0.3%. Hence, the implementation of a pinhole array with LED illumination can produce multi-focus with uniform intensity and constant pitch. Table S1 details the components used to build the MC-ISM, with a total cost of only RMB 31 858. Additionally, the implementation steps for the optical path have been detailed on GitHub, and the LabVIEW synchronous code, including 1D and 2D scanning for the galvanometer, has been open-sourced.

### PR reconstruction with OLID preprocessing

**Figure 2.**
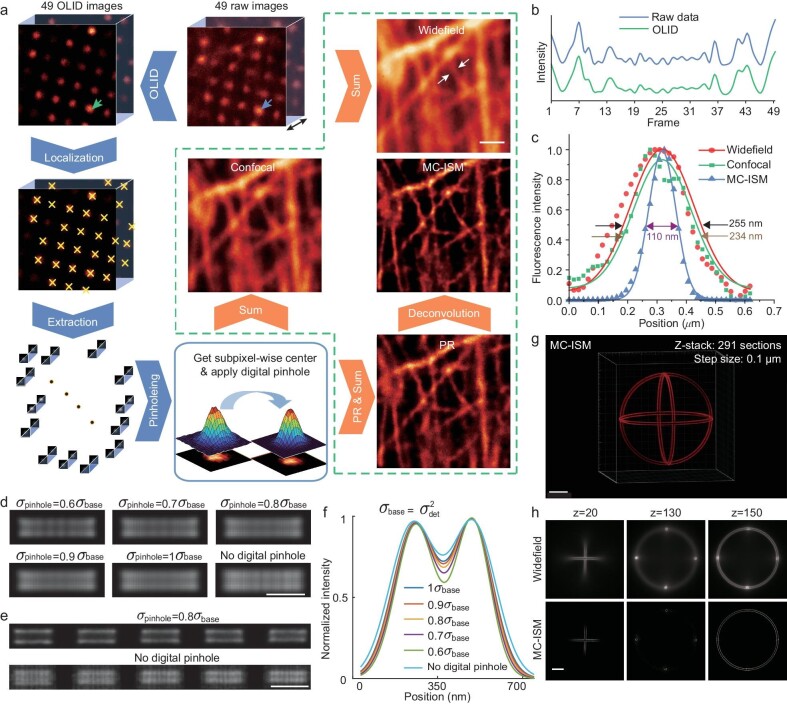
Pixel reassignment super-resolution reconstruction process and reconstruction results. (a) Flow chart of the reconstruction algorithm, including OLID preprocessing, the pixel-level center positioning, extraction of sub-images, applying digital pinholes, and pixel reassignment. Scale bar: 1 μm. (b) Comparison of OLID processing results with raw images. (c) Resolution measurements for widefield, confocal and MC-ISM. (d) MC-ISM reconstruction results of 140 nm spacing line pair under Gaussian digital pinholes with different widths (standard deviations). Scale bar: 0.5 μm. (e) After adding Gaussian noise to make raw images SNR 10 dB, MC-ISM reconstruction results of line pair from 170 nm to 210 nm with and without digital pinhole, respectively. Scale bar: 1 μm. (f) Intensity distribution of line pairs in Fig. 2d. (g) Three-dimensional display of the 3D sphere with a diameter of 25 μm. Scale bar: 5 μm. (h) Image results of widefield and MC-ISM of a 3D sphere with a diameter of 25 μm at different z slices. Scale bar: 5 μm.

The comprehensive overview of the PR reconstruction process is shown in Fig. 2a and Note S3), which consists of the following steps: (1) OLID preprocessing, (2) positioning the pixel-level center, (3) extracting sub-images, (4) applying digital pinholes, and (5) pixel reassignment. The MC-ISM result can be obtained through the deconvolution of PR.

Pixel reassignment is contingent upon the accurate extraction of each illumination spot, however, when the in-focus signals are weak, it can easily drown in the out-of-focus background. We incorporate OLID to enhance the accuracy of positioning the illumination spots in scattering samples by removing out-of-focus signals. The intuition of OLID is regarding the multi-focus scanning utilized by MC-ISM as a spatial modulation. The in-focus signals demonstrate prominent alternating current (AC) characteristics owing to the modulation of the illumination pattern, while the out-of-focus signals remain constant during scanning, exhibiting direct current (DC) characteristics. As shown in Fig. S5, the raw data undergoes Fourier transformation pixel by pixel along the modulation direction, where the high frequencies mainly contain the modulated AC components, and the zero frequency contains both the entire DC and AC components. Hence, employing OLID to extract modulation frequency components and attenuate the zero-frequency component along the modulation direction can effectively eliminate the out-of-focus signals [27] (Fig. 2b).

Subsequently, a sub-image was cropped based on the position of the illumination spot, and PR was achieved by displacing the center of the sub-image to double the original distance. It has been demonstrated that even if there is a slight deviation between the located center and the actual center of the illumination spot, as long as the sub-image encompasses the entire illumination spot, the PR result remains unaffected. The error caused by pixelation, which only allows the image to be displayed at integer indices, can be mitigated by upsampling the raw image (Fig. S6).

Combining PR with OLID and balancing removal of out-of-focus signals and imaging speed, a pinhole array with a 1:3 ratio of diameter and pitch can be employed. In addition, it has also been experimentally proven that large pitch can more effectively reduce the out-of-focus signal in original images (Fig. S7). A larger pinhole distance can be used to further remove out-of-focus signals when imaging highly scattering thick specimens.

We imaged fixed actin filaments using a 40 μm:120 μm (pinhole diameter:pitch) pinhole array and performed widefield, confocal, and MC-ISM reconstruction with the original 49 images, a 110 nm resolution can be reached by MC-ISM (680 nm emission, Fig. 2c), doubling the resolution attainable from widefield imaging (255 nm).

Simulations of the digital pinhole effect were conducted. The digital pinhole, represented as a Gaussian mask centered on the illumination spot within each sub-image, plays a pivotal role in the PR reconstruction process. The MC-ISM reconstruction results of line pairs spaced at 140 nm with different digital pinhole size are shown in Fig. 2d. As the digital pinhole size decreases, line pairs become more discernible. Fig. 2f shows the resolution improvement from a quantitative perspective by the Bi-Gaussian fitting. However, as the digital pinhole size decreases to $0.6{\sigma }_{{\mathrm{base}}}$, the MC-ISM reconstruction results exhibit discontinuities. Simultaneously, Gaussian noise is intentionally introduced into the original images to reduce the signal to noise ratio (SNR) to 10 dB. As shown in Fig. 2e, application of a digital pinhole can effectively enhance resolution and SNR while mitigating reconstruction artifacts.

The resolving ability of MC-ISM was also verified by imaging and reconstructing the fluorescent beads with a diameter of 40 nm. The reconstruction result of MC-ISM showed higher 3D resolution compared to widefield (Fig. S8a), with lateral resolution reaching 130 nm and axial resolution reaching 423 nm (680 nm emission, Fig. S8b).

A 3D sphere with a diameter of 25 μm (ArgoSIM, Argolight) was reconstructed to demonstrate the 3D reconstruction accuracy of MC-ISM (imaging depth: 29 μm, step interval: 0.1 μm). The 3D structure of the sphere is displayed (Fig. 2g), with the comparative results between the widefield and MC-ISM in the three layers shown in the Fig. 2h (Movie S2). The enhancement in 3D resolution enables a distinct differentiation of the dual-layer outline constituting the entire sphere.

### Frame reduction reconstruction utilizing redundant information in ISM

**Figure 3.**
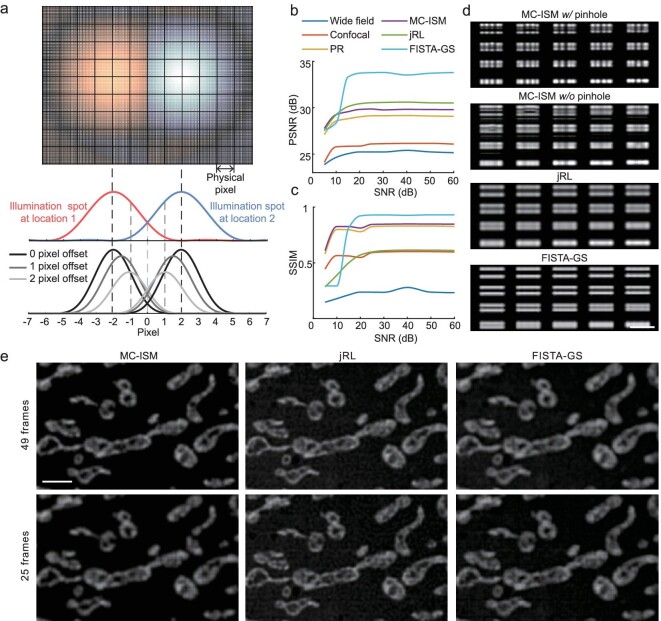
Super-resolution frame reduction reconstruction using a multi-image deconvolution algorithm. (a) Top: Schematic diagram of the simulation at different positions of the scanning optical axis, and the camera pixels occupied by the 1AU spot. Bottom: Simulation results of PSF of the system detected by the camera pixels shifted by different distances compared to the optical axis of the illumination (zero-pixel offset). (b and c) As the SNR of the original image increases, the SSIM and PSNR values for the reconstructed images using various methods change. (d) Reconstruction results of different methods with a scanning step of 0.5 AU. Scale bar: 1 μm. (e) In the case of fixed pinhole array pitch, frame reduction reconstruction results of jRL and FISTA-GS, with MC-ISM reconstruction results as a comparison. Scale bar: 2 μm.

ISM utilizes array detection, where each pixel functions akin to a point detector, thereby having much richer information over confocal microscopy [25,34]. We reduce the number of frames by enlarging the scanning step size, which could lead to gaps within the PR reconstruction. Nonetheless, there remains a degree of overlap among the illumination spots at adjacent scanning locations, indicating comprehensive excitation of the sample area with varying intensities (Fig. S9a). We probed the effective PSF of detection pixels at different offsets from the excitation optical axis (Fig. 3a), to evaluate how distinct pixels capture sample information. Our findings reveal that each pixel's effective PSF exhibits a certain degree of overlap with its adjacent pixel's effective PSF. This signifies that during image formation, a given pixel detects signals emanating from diverse sample positions (Fig. S9b), and any particular sample location is repeatedly detected by multiple pixels (Fig. S9c). Employing appropriate multi-image deconvolution algorithms can help to pick intensities from the observed image and re-assign them to achieve higher quality reconstruction results. Joint Richardson-Lucy (jRL) is one of the most common multi-image deconvolution algorithms for the reconstruction of MSIM [26,35]. Here we propose to combine OLID with jRL in strong-background and low-SNR conditions for artifacts-mitigation (Fig. S10).

Considering jRL cannot uniformly improve resolution for different structures and relies on the model assumption that the noise follows Poisson distribution, we developed a deconvolution method called FISTA-GS to achieve fidelity reconstruction [29]. It reconstructs ${O}_i( r )$ by solving the following optimization problem:


\begin{eqnarray*}
{O}_i &=& \mathop {\arg \min }\limits_{{O}_i} \left[ \frac{1}{2}\sum\limits_i {\left\| {{I}_i\left( r \right) - {I}_{i,{\mathrm{noise}}}\left( r \right)} \right\|} _2^2\right.\\
&&\qquad \qquad\left. + \lambda {{\left\| {\sum\limits_i {{O}_i\left( r \right)} } \right\|}}_1 \right],
\end{eqnarray*}

where ${I}_i( r )$ is the image of the solution target ${O}_i( r )$. The image formation of ${I}_i( r )$ from ${O}_i( r )$ is modeled as first modulated by the illumination pattern and then convolved by the PSF. The first term on the right-hand side is the fidelity term to ensure that ${I}_i( r )$ is close to the measured image ${I}_{i,{\mathrm{noise}}}( r )$, and the second term is the group sparsity regularization term. Detailed explanation and the implementation process of jRL and FISTA-GS is given in Note S4. We compared the performance of the MSIM reconstruction algorithm with our reconstruction algorithm (PR, jRL and FISTA-GS) using the open-source dataset included with MSIM [23] (Fig. S11). Compared with the MSIM reconstruction, microtubules reconstructed by MC-ISM have less background and can be resolved more clearly in overlapping areas (neither MSIM nor MC-ISM further deconvolves the image). Results reconstructed by jRL and FISTA-GS achieve comparable resolution without edge clipping. The reconstruction result of jRL shows a finer microtubule structure, while FISTA-GS shows a better SNR and structural continuity.

Various reconstruction methods, including widefield, confocal, pixel reassignment, MC-ISM, jRL and FISTA-GS, are systematically evaluated by simulation (Fig. S12 and Note S5). It turned out that FISTA-GS emerged as the frontrunner in both peak signal-to noise-ratio (PSNR) and structural similarity (SSIM) metrics when no noise was added (Table S2), effortlessly distinguishing 140 nm line pairs (Fig. S12a). When Gaussian and Poisson noise were deliberately introduced to make the SNR of raw images vary from 5 dB to 25 dB, the reconstruction performance of these methods all tended to be stable when the acquired image quality exceeded a SNR of 25 dB. In such a case, FISTA-GS exhibited the highest PSNR and SSIM values (Fig. 3b, c and Fig. S12b). Additionally, the line pairs reconstructed by pixel reassignment exhibited discontinuities when the scanning step increases to 0.5 AU. It is noteworthy that neither jRL nor FISTA-GS exhibited this discontinuity. Therefore, both FISTA-GS and jRL show the capability of reconstruction under a low sampling ratio (Fig. 3d). jRL and FISTA-GS with 25 frames show similar reconstruction performance with MC-ISM with 49 frames in fixed mitochondria samples (BPAE cells), and FISTA-GS exhibits a higher fidelity compared to jRL (Fig. 3e). The number of frames was reduced from 49 to 25, and at a tilt angle of 11.1°, the scanning step size increased by 1.39 times (Note S1). Consequently, it is feasible to attain a two-fold enhancement in imaging speed by leveraging these two algorithms. The PR and multi-image deconvolution algorithms, along with detailed reconstruction instructions, have been made publicly available on GitHub.

### Super-resolution imaging of biological tissues

**Figure 4.**
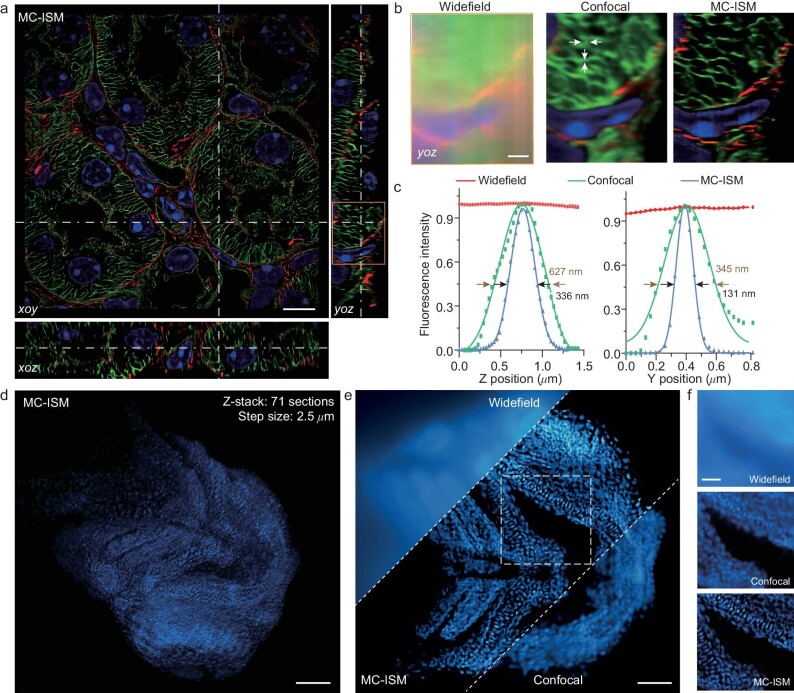
The applicability of MC-ISM to tissue samples. (a) Orthogonal display of 4D imaging results of mouse kidney section in MC-ISM system. Imaging depth: 12 μm. Scale bar: 10 μm. (b) The magnified results of the orange-line box in Fig. 4a are depicted using widefield, confocal, and MC-ISM. Scale bar: 2 μm. (c) The pixel intensity indicated by white arrows along the y and z directions in Fig. 4b. (d) 3D display of a DAPI-stained zebrafish head imaged with a long working distance 20 × air objective. The total imaging depth is 175 μm, measured from the location where the fluorescent signals are generated. Scale bar: 70 μm. (e) Zebrafish head compared for widefield, confocal, and MC-ISM. Scale bar: 70 μm. (f) Comparison of three imaging modalities of zebrafish in the area indicated by the white-dashed box in Fig. 4e. Scale bar: 25 μm.

To demonstrate the 3D super-resolution imaging of tissue samples, we performed tri-color 3D imaging of a mouse kidney section in a volume of 66.5 μm × 66.5 μm × 12 μm at an axial interval of 150 nm through a 40 μm:200 μm pinole array (Fig. 4a and Movie S3). Notably, the filamentous actin, elements of the nephron, and nuclei structure are distinctly separated (Fig. 4b), with a 2-fold resolution improvement in both lateral (131 nm) and axial (336 nm) dimensions (Fig. 4c). MC-ISM effectively rejects out-of-focus signals and attains comparable optical sectioning performance with LiveSR super-resolution spinning disk confocal (SR-SD) and Airyscan microscope (Fig. S13). The resolutions achievable with the three imaging methods under the same sample were compared using Fourier ring correlation [36], resulting in 135 nm (MC-ISM), 165 nm (SR-SD) and 146 nm (Airyscan), respectively. Additionally, a performance radar chart was created based on public data and product manuals to illustrate the imaging advantages of MC-ISM more clearly (Fig. S14, Table S3 and Note S6).

MC-ISM is also compatible with a low magnification objective to attain superior imaging depth. The depth imaging of a DAPI-stained zebrafish head was performed using a 20 × objective with a long working distance and a 40 μm:280 μm pinhole array in a volume of 665 μm × 665 μm × 175 μm at an axial interval of 2.5 μm. The effective removal of out-of-focus signals across the entire volume is highlighted in Fig. 4d (Movie S4) which enables clear observation of individual cells. Particularly, at z = 90 μm, MC-ISM proficiently eradicates out-of-focus signals at considerable depth compared to widefield and confocal techniques which achieve improvement in resolution (Fig. 4e and f).

### Mitochondrial dynamic super-resolution imaging

**Figure 5.**
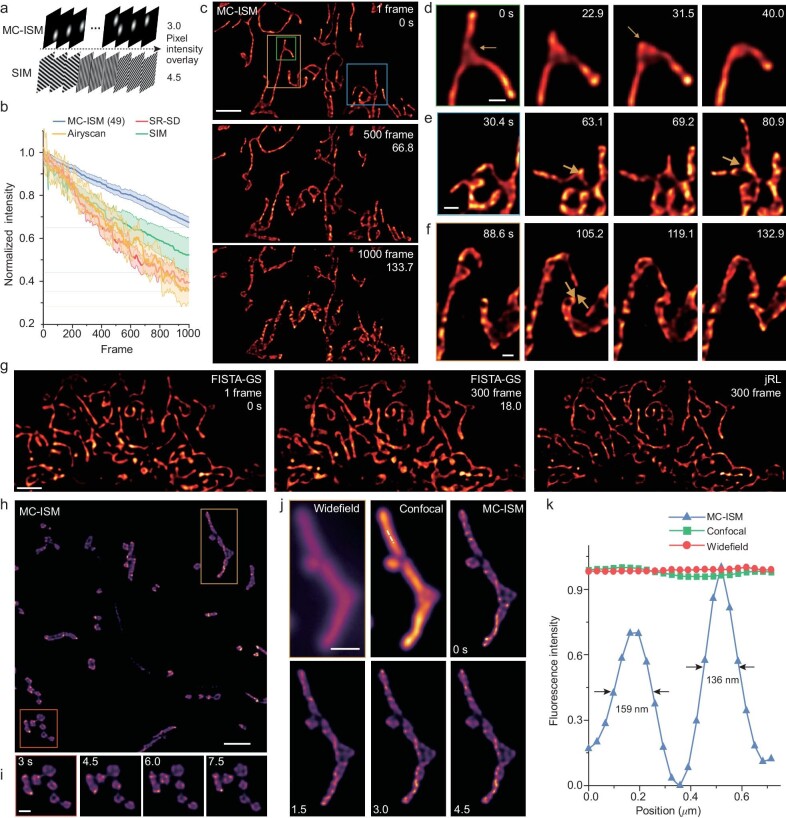
MC-ISM super-resolution dynamic observation of mitochondria in living animal cells and plant tissues. (a) A comparative analysis of illumination light dose between MC-ISM and SIM. (b) Comparison of photobleaching between the MC-ISM system and different commercial systems (LiveSR super-resolution spinning disk confocal (1.7× magnification module), Zeiss LSM 980 with Airyscan 2 (Airyscan acquisition mode), and GE Healthcare DeltaVision OMX 3D-SIM (2D SIM acquisition)). (c) Start and end frames of the 1000-frame stack of live mitochondria labeled with HBmito Crimson. Scale bar: 3 μm. (d) The mitochondrial branch retracts from the branch point in the green-line box of Fig. 5c. Scale bar: 0.5 μm. (e) Mitochondria elongate new branches in the blue line box of Fig. 5c. Scale bar: 0.5 μm. (f) Fusion of two mitochondria in the dark-yellow line box of Fig. 5c. Scale bar: 1 μm. (g) Frame reduction reconstruction results of jRL and FISTA-GS of live cell mitochondria. Scale bar: 3 μm. (h) MC-ISM Super-resolution imaging result of HBmito Crimson–labeled Arabidopsis hypocotyls mitochondria. Imaging depth: 20 μm. Scale bar: 10 μm. (i) Dynamic imaging of spherical mitochondria in the red line box of Fig. 5h. Scale bar: 0.5 μm. (j) Dynamic imaging of elongated mitochondria cristae in the yellow line box of Fig. 5h, with a comparison of widefield, confocal and, MC-ISM at 0 seconds. Scale bar: 1 μm. (k) Pixel intensity of the white dashed lines along the mitochondrion in Fig. 5j.

MC-ISM shows enhanced photon efficiency owing to its simplified detection light path. Considering the impact of spot superposition during scanning at various positions, the light dose irradiated on the sample is only 2/3 of that of SIM [8] (Fig. 5a). When considering equivalent numbers of reconstructed frames and SNRs, the photobleaching by MC-ISM (49 raw images) and different commercial microscopes was compared using fixed actin filaments labeled by Phalloidin-Atto 647N. The light source power and exposure time were adjusted so that the SNR of images collected by different microscopes were basically at the same level (MC-ISM, SIM and Airyscan are the average overlay of all raw data). On this basis, the intensity attenuation after 1000 frames of each microscopy was compared to evaluate the photobleaching. After 1000 frames of time-lapse imaging, the fluorescence intensity of MC-ISM drops to 67%, and the photobleaching is lower than OMX SIM (52%), SR-SD (39%) and Airyscan (36%). Therefore, the light dose irradiated by MC-ISM on the sample is the lowest when considering equivalent numbers of reconstructed frames and SNRs (Fig. 5b).

Mitochondria are highly dynamic organelles, with the distance between cristaes typically <120 nm. In addition, mitochondria are very light-sensitive and extremely susceptible to swelling due to phototoxicity, which makes them a highly suitable target organelle for evaluating phototoxicity of super-resolution imaging systems. The imaging of the mitochondria in U2OS cells labeled with HBmito Crimson was performed at a speed of 7.5 frames per second (fps) (Fig. 5c and Movie S5). In the observation window of 1000 frames, the mitochondrial cristae structure was resolved, and the mitochondria did not swell. During this process, cristae at sites of mitochondrial contraction appear sparser (Fig. 5d), suggesting that cristae remodeling is less stressful in these sites. Also, the newly extended branch cristae are relatively sparse (Fig. 5e), which might be because the inner membrane arrangement of newly generated branches has not been completed. Additionally, the mitochondrial fusion process was captured (Fig. 5f). FISTA-GS and jRL were also employed for reconstruction, utilizing 25 frames of raw data to achieve high-speed imaging in COS7 cells labeled with HBmito Crimson at 16.7 fps. It is evident that the FISTA-GS reconstruction yields significantly superior results compared to jRL during fast imaging (Fig. 5g). The excellent resolution and extremely low phototoxicity of the MC-ISM system provide a suitable tool for the study of mitochondrial dynamics.

The mitochondrial inner membrane imaging of animal cells has been widely studied due to the advancement of super-resolution microscopy, while plant cells are difficult to image in depth due to tissue scattering and autofluorescence. The advantage of removing out-of-focus signals and 2-fold enhancement in the 3D resolution of MC-ISM makes it the perfect tool for *in vivo* super-resolution imaging of mitochondria in thick plant tissue. The *in vivo* imaging of mitochondria in Arabidopsis hypocotyls was performed using a 40 μm:160 μm pinhole array to observe the inner membrane structure (Fig. 5h). It is observed that besides the elongated mitochondria similar to animal cells with cristae distributed perpendicular to the direction of its extension (Fig. 5j), there are also spherical mitochondria in plant cells with sheet-like cristae distributed in the mitochondrial matrix (Fig. 5i) [37]. In addition, the spacing between mitochondrial cristae clusters in plant cells is notably greater compared to that in animal mitochondria. This disparity might be attributed to the reduced requirement for a larger inner membrane area in plants due to their lower energy supply demands. Our observations reveal that spherical mitochondria exhibit a rapid completion of cristae remodeling, potentially attributed to their smaller size, which facilitates the remodeling process. Conversely, elongated and larger mitochondria exhibit constrained alterations in cristae morphology within a 4.5 seconds timeframe (Fig. 5j and i). We also observed the complete fusion process of plant mitochondria, as shown in Fig. S15. Via resolution measurements, MC-ISM demonstrates a remarkable lateral resolution of 136 nm even within plant tissue (Fig. 5k), underscoring its broad applicability across diverse sample types.

## DISCUSSION

MC-ISM emerges as an advantageous tool for *in situ* imaging of subcellular dynamics within biological tissues, markedly elevating imaging speed compared to MSIM [23], from 1 fps to 16.7 fps (Table S4). The enhancement in imaging speed stems from advancements in three aspects: (1) the generation and stepping of multi-focus are performed independently using two different devices. The DMD used in MSIM is coupled on the pinhole size and the step interval. In order to meet the Nyquist sampling step, the equivalent excitation pinhole size of the DMD is 120 nm, resulting in increasing scanning step. (2) OLID and multi-focus scanning methods are ingeniously integrated to enhance the precision of illumination point positioning in scattered samples by eliminating out-of-focus signals. Therefore, our pinhole array pitch can be smaller when interfered by the same out-of-focus signals, thereby improving imaging speed. (3) Multi-image deconvolution methods are utilized to reconstruct with fewer frames, resulting in acceleration of imaging speed.

Digital reconstruction technology exhibits lower phototoxicity which can be attributed to its straightforward detection optical path. Even after 1000 consecutive frames of imaging of mitochondria in living cells, no obvious swelling of mitochondria was seen. Pixel reassignment reconstruction with OLID preprocessing achieves a lateral resolution of 131 nm and an axial resolution of 336 nm on three-dimensional tissue samples, and the imaging depth reaches 175 μm under a 20 × objective lens. In addition, the implementation of a pinhole array with LED illumination can produce multi-focus with uniform intensity and constant pitch within a FOV of 100 μm. This capability enables MC-ISM to perform *in situ* imaging in scattering biological tissues.

One of the features of MC-ISM is its flexibility in adapting to a wide range of biological specimens. This adaptability is primarily enabled by the free choice of the aspect ratio of the pinhole arrays used in the system, making MC-ISM highly compatible with different biological samples. Another key advantage of MC-ISM is its insensitivity to the light source used. By utilizing incoherent light sources, the system effectively mitigates issues related to speckle, resulting in more uniform and high-quality illumination across a large FOV. The consistent and uniform illumination of MC-ISM is critical for accurate analysis in different imaging applications. Additionally, MC-ISM offers a cost-effective solution for high-quality imaging. It can be seamlessly integrated into existing confocal microscopy setups, simply by replacing a single pinhole with a pinhole array and a single-point detector with a camera.

The scanning step of galvanometers is not stable for small-angle scanning since they are typically engineered for large-angle and rapid scanning applications [32]. To address the instability associated with small-angle step signal inputs, the feedback circuit's output characteristics were adjusted to align with the desired motion trajectory. In the case of employing two-dimensional galvanometers for scanning, particularly for the X-axis mirror, precise and rapid retracing at small angles is essential. However, due to inertia, the mirror movement displays nonlinearity during the initial stages of scanning, which is difficult to be mitigated through voltage correction methods. When employing a single galvanometer for scanning, extending the scanning length by a factor of 7 has notably mitigated these nonlinear effects, and the generation of artifacts from the scanning is avoided.

In terms of the reconstruction algorithm, the resolution improvement of MC-ISM can be understood not only through pixel reassignment in the spatial domain, but also through the illumination pattern as a form of structured illumination like SIM (Note S7), which can be easily analyzed in the frequency domain. Based on such ideas, the forward propagation model of MC-ISM is established and applied into joint deconvolution methods. Another innovation of the algorithm is utilizing OLID to remove out-of-focus signals for artifact-free reconstruction, and FISTA with group sparsity constraints to iteratively obtain fidelity super-resolution images.

One of the most exciting breakthroughs facilitated by MC-ISM is its ability to reveal the inner membrane structure of plant mitochondria for the first time. This demonstrates the microscope's exceptional capabilities in capturing fine details and previously inaccessible information within biological specimens. This capability can bring a profound impact on various fields, from cell biology to plant science. The microscope's compatibility with existing confocal systems and its cost-effectiveness makes it a promising contender to usher in the next era of confocal microscopy.

## MATERIALS AND METHODS

For detailed materials and methods, please see the supplementary data.

### Hardware implementation

The MC-ISM system was mounted on a Nikon inverted fluorescence microscope (Nikon Ti2-E). Except for the zebrafish experiment, which used a 20 × objective lens (CFI S Plan Fluor LWD ADM 20 × 0.7NA, Nikon), the rest of the experiments used a 100 × objective lens (CFI SR HP Apo TIRF 100 × 1.49NA, Nikon). An 8-independently-controllable integrated light source was used (SPECTRA Light Engine, Lumencor) with multi-band dichroic mirrors and emitters (89 402 Multi LED set, Chroma) for multi-color (DAPI, GFP, Cy3 and Cy5) imaging. High-power LED transmitted through Ø3 mm core liquid light guide was collimated using an aspheric condenser lens (MAC4606-A, LBTEK) to provide illumination with a large FOV. An LED was passed through a pinhole array etched onto chrome glass to produce multifocal illumination. A pair of galvanometers (S-8107, Sunny Technology) was used to make the multi-focus illumination points uniformly scan the sample, and the scan lens (AC254-75, Thorlabs) and the tube lens (200 mm, Nikon) conjugated the center of the galvanometer to the entrance pupil of the objective. Under small angle (0.2°) scanning, the switching speed of the galvanometer can reach 6.7 kHz. A single lens reflex (SLR) lens (105 mm F2.8 MACRO, SIGMA) was connected to the sCMOS (ORCA-Flash4.0 V3, Hamamatsu) as a 1:1 relay lens to achieve large FOV imaging.

### Instrument control

The microscope achieved synchronous control of all equipment through 3 analog signals and 2 digital signals output by the data acquisition card (USB-6363, NI) and Labview (NI) programming. One analog signal was used to control the scanning of the galvanometer, another analog signal was used for the external trigger of the sCMOS, and the last analog signal was used for the control of the piezo sample scanner. Two digital signals were used to achieve synchronization and arbitrary switching of the light sources.

### Deconvolution algorithm

The data reconstructed through PR can be further enhanced in resolution through deconvolution. The data presented in the manuscript were processed using Huygens (SVI, Netherlands) with default parameters. Additionally, through testing, we found that the open-source deconvolution algorithm, Multi-Resolution Analysis Deconvolution [38], can achieve similar results (Fig. S16).

### Live cell imaging

Cells were cultured on confocal dishes 24 h before the experiments at 37°C in a 5% CO_2_ atmosphere with 95% humidity. Before imaging, cells were incubated with HBmito Crimson [33] (mitochondrial inner membrane probe, excitation wavelength: 640 nm) for 10 min at 500 nM concentration, and the live cell incubation chamber was used to maintain the cells in an environment of 37°C and 5% CO_2_.

### Arabidopsis culture and mitochondrial inner membrane labeling

Arabidopsis seeds were germinated on 1/2 MS (Murashige and Skoog) agar plate. After being kept at 4°C for 1 day, seeds were cultured under 21°C with 16 h light and 8 h dark. The seeds were cultured for 7–12 days. The hypocotyl of around ten-day-old Arabidopsis seedlings was incubated overnight in 1 μM of HBmito Crimson at room temperature. Before microscopy imaging, the hypocotyl was incubated in 5 μM of dye for observation.

## Supplementary Material

nwae303_Supplemental_Files

## Data Availability

The algorithm reconstruction codes, example raw data, hardware control code and user's guide are available at the GitHub repository (https://github.com/Chauncey-Leung/MC-ISM).
