# Supplementary material for: Expanding super-resolution imaging versatility in organisms with multi-confocal image scanning microscopy
Source: Natl Sci Rev. 2024 Aug 27;11(9):nwae303. doi: 10.1093/nsr/nwae303 (PMC11879394; doi:10.1093/nsr/nwae303)
Supplement: nwae303_Supplemental_Files [file nwae303_supplemental_files.zip › Supporting Information.pdf]

Supporting Information

Expanding super-resolution imaging versatility in organisms with multi-confocal image scanning microscopy

Wei Ren[1,2,†], Meiling Guan[1,2,6,†], Qianxi Liang[1,2,†], Meiqi Li[3,*], Boya Jin[1,2], Guangxing Duan[3], Liya Zhang[3], Xichuan Ge[4], Hong Xu[5], Yiwei Hou[1,2], Baoxiang Gao[4], Sodmergen[3], Peng Xi[1,2,*]

Corresponding author: P. X. (xipeng@pku.edu.cn) or M. L. (limeiqi@pku.edu.cn)

Table of content:

Materials and Methods……………………………………………….……………3

Supplementary Figures 1 to 16……………………………………………………..…6

Table S1 List of components used in the MC-ISM……………………………….......24

Table S2 Comparison of different reconstruction methods for noise-free images…....25

Table S3 Comparison of Airyscan, SR-SD and MC-ISM implementation methods....26

Table S4 Comparison of related technical performance based on ISM………………27

Supplementary Notes 1 Comparison of 1D and 2D scanning……………………......28

Supplementary Notes 2 Simulations showing the effect of different excitation pinhole diameters, scan steps, and digital pinhole sizes on MC-ISM results. …………………30

Supplementary Notes 3 MC-ISM reconstruction in the spatial domain. …………....32

Supplementary Notes 4 The implementation process of jRL and FISTA-GS…..…..34

Supplementary Notes 5 Resolution and image quality assessment for multiple reconstruction methods. ……………………………………………………………...40

Supplementary Notes 6 Performance comparison of Airyscan, SR-SD and MC-ISM. ……………………………………………………………………………….…42

Supplementary Notes 7 MC-ISM imaging formation model….……………………44

References……………………………………………………………………...47

Other supporting materials for this manuscript include the following:

Movie S1. The generation process of raw images, OLID images and PR stacking in one-dimensional scanning.

Movie S2. The display of the three-dimensional sphere with a diameter of 25 μm.

Movie S3. The four-dimensional imaging result of MC-ISM for mouse kidney section.

Movie S4. The three-dimensional imaging result of MC-ISM for a zebrafish head.

Movie S5. The time-lapse imaging result of mitochondria in living cells reconstructed by PR.

MATERIALS AND METHODS

Hardware implementation

The MC-ISM system was mounted on a Nikon inverted fluorescence microscope (Nikon Ti2-E). Except for the zebrafish experiment, which used a 20× objective lens (CFI S Plan Fluor LWD ADM 20× 0.7NA, Nikon), the rest of the experiments used a 100× objective lens (CFI SR HP Apo TIRF 100× 1.49NA, Nikon). An 8-independently-controllable integrated light source was used (SPECTRA Light Engine, Lumencor) with multi-band dichroic mirrors and emitters (89402 Multi LED set, Chroma) for multi-color (DAPI, GFP, Cy3 and Cy5) imaging. High-power LED transmitted through Ø3 mm core liquid light guide was collimated using an aspheric condenser lens (ACL2520U-A, Thorlabs) to provide illumination with a large FOV. LED pass through a pinhole array etched on chrome glass to produce multifocal illumination. A pair of galvanometers (S-8107, Sunny Technology) was used to make the multi-focus illumination points uniformly scan the sample, and the scan lens (AC254-75, Thorlabs) and the tube lens (200mm, Nikon) conjugated the center of the galvanometer to the entrance pupil of the objective. Under small angle (0.2°) scanning, the switching speed of the galvanometer can reach 6.7KHz. A single lens reflex (SLR) lens (105mm F2.8 MACRO, SIGMA) was connected to the sCMOS (ORCA-Flash4.0 V3, Hamamatsu) as a 1:1 relay lens to achieve a large FOV imaging. The main body of the microscope was equipped with a nano-positioning piezo sample scanner (NanoScan SP400, Prior) and a live cell incubation chamber (OKOLab) to achieve z-axis scanning and live cell imaging.

Instrument control

The microscope achieved the synchronous control of all equipment through 3 analog signals and 2 digital signals output by the data acquisition card (USB-6363, NI) and Labview (NI) programming. One analog signal was used to control the scanning of the galvanometer, another analog signal was used for the external trigger of the sCMOS, and the last analog signal was used for the control of the piezo sample scanner. Two digital signals were used as addressing codes to cooperate with the sCMOS “Global exposure timing output (Negative active)” to achieve synchronization and arbitrary switching of the light sources through the demultiplexer. We used the “Camera Link data output” time in the sCMOS “Global reset edge trigger mode” to realize the

stepping and stabilization of the galvanometer. The time sequence of single-frame acquisition and five-dimensional acquisition (XYCZT) is shown in Supplementary Fig. 3.

Evaluation of illumination pattern's distortion

Distortion is a type of geometrical aberration that causes misplacement of points relative to the center of the field, resulting in uneven distribution of the multi-focus illumination pattern. Distortion is calculated by:

$$D(\%) = \frac{y_a - y_p}{y_p} \times 100\%$$

Where y_a is the actual distance and y_p is the predicted distance. We use the average of the point spacing in the central FOV (the white box in the middle in Fig. 1e) to represent y_a, and that of the edge FOV (the four surrounding white boxes in Fig. 1e) to represent y_p approximately.

SNR measurement

The displacement correction was performed on the first 100 frames of the collected 1000 frames, by randomly selecting 10 regions of interest and obtaining the normalized intensity distribution curves. Baseline fitting was then performed by linear regression, and the fitted baseline was subtracted from the raw data to compensate for intensity loss due to photobleaching, and its mean and standard deviation were calculated to obtain the SNR.

Sample preparation

Live cell imaging

U2OS cells were cultured at a suitable density (moderate) in DMEM (Gibco) supplemented with 10% (v/v) fetal bovine serum (FBS, Gibco), 100 units/mL penicillin and 100 mg/mL streptomycin (PS, Gibco) at 37°C in a 5% CO2 atmosphere with 95% humidity. Cells were cultured on confocal dishes 24 h before the experiments. Before imaging, cells were incubated with HBmito Crimson[1] (mitochondrial inner membrane probe, excitation wavelength: 640 nm) for 10 min at 500 nM concentration,

and the live cell incubation chamber was used to maintain the cells in an environment of 37°C and 5% CO_2.

Fixed actin filaments

U2OS cells were fixed with 4% formaldehyde (FB002, Thermo) for 10 min, then washed three times with PBS to remove the formaldehyde. Phalloidin-Atto 647N (65906, Sigma) was used to stain the actin filaments for 1 h at room temperature. Then, the coverslip was sealed on the slide with the prolonged antifade mountant (P36965, Thermo).

Fixed mitochondria

The fixed mitochondria sample used to show the effect of frame reduction reconstruction (Fig. 3e) was purchased from ThermoFisher (FluoCells #1, F36924).

Mouse kidney sections

The mouse kidney section used for 3D imaging (Fig. 4a and 4b) was purchased from ThermoFisher (FluoCells #3, F24630).

Zebrafish culture and preparation

The zebrafish (three days post fertilization) were anesthetized with Tricaine, and placed in a confocal dish. Then the zebrafish was embedded into a 1.5% low melting point agarose to make it as close as possible to the coverslip. At the same time, adjust the posture so that the head is downward, and wait for the agarose gel to solidify before imaging.

Arabidopsis culture and mitochondrial inner membrane labeling

Arabidopsis seeds were germinated on 1/2 MS (Murashige and Skoog) agar plate. After keeping at 4°C for 1 day, seeds were cultured under 21°C with 16-h light and 8-h dark. The seeds were cultured for 7-12 days. The hypocotyl of about ten-day-old Arabidopsis seedlings was incubated overnight in 1 μM of HBmito Crimson at room temperature. Before microscopy imaging, the hypocotyl was incubated in 5 μM of dye for observation.

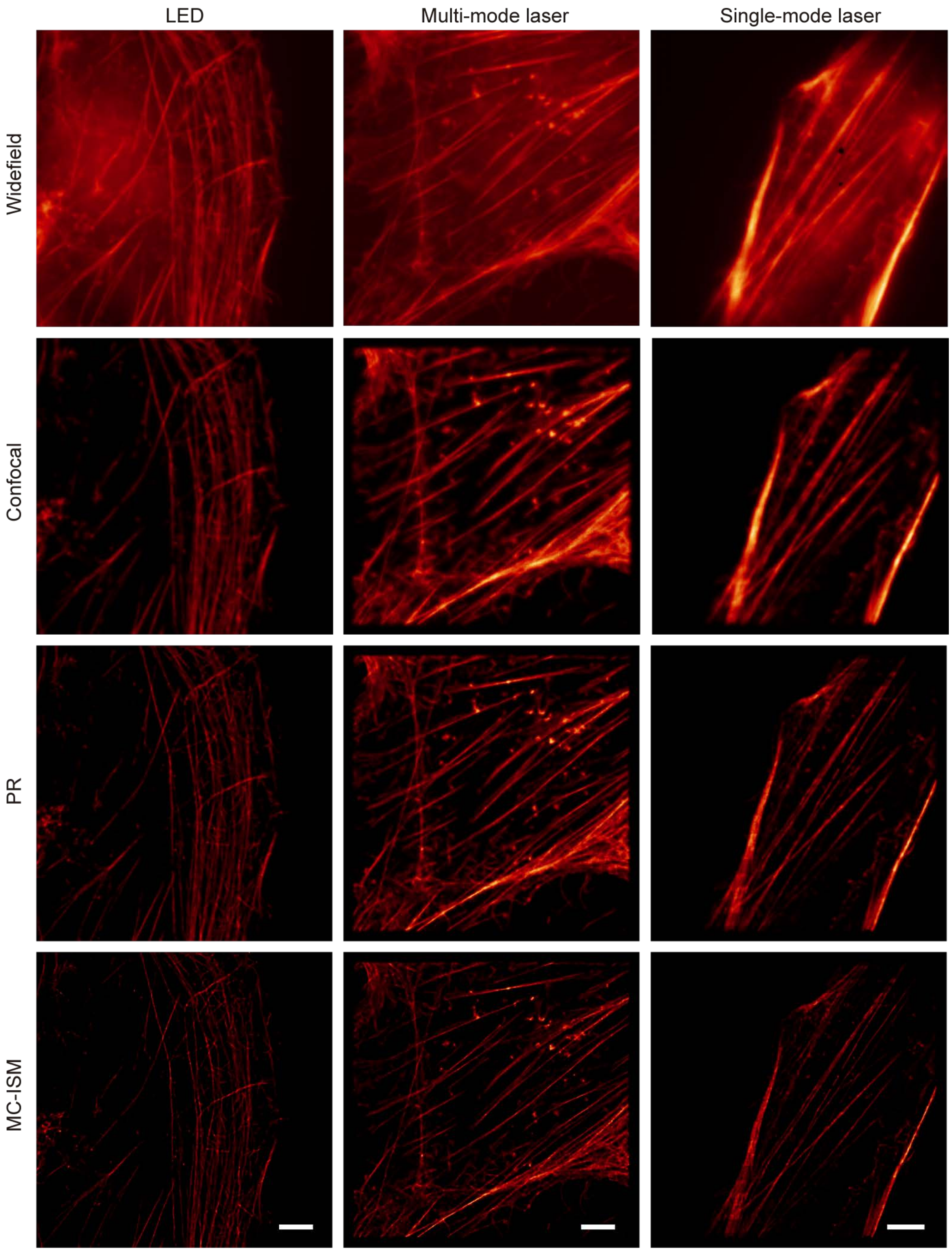

Supplementary Fig. 1. Comparison of imaging results of widefield, confocal, PR, and MC-ISM systems illuminated by Lumencor SPECTRA Light Engine LED source, 89 NORTH laser diode illuminator (LDI) multi-mode laser, and COHERENT OBIS 637nm single-mode laser. Scale bar: 4 μm.

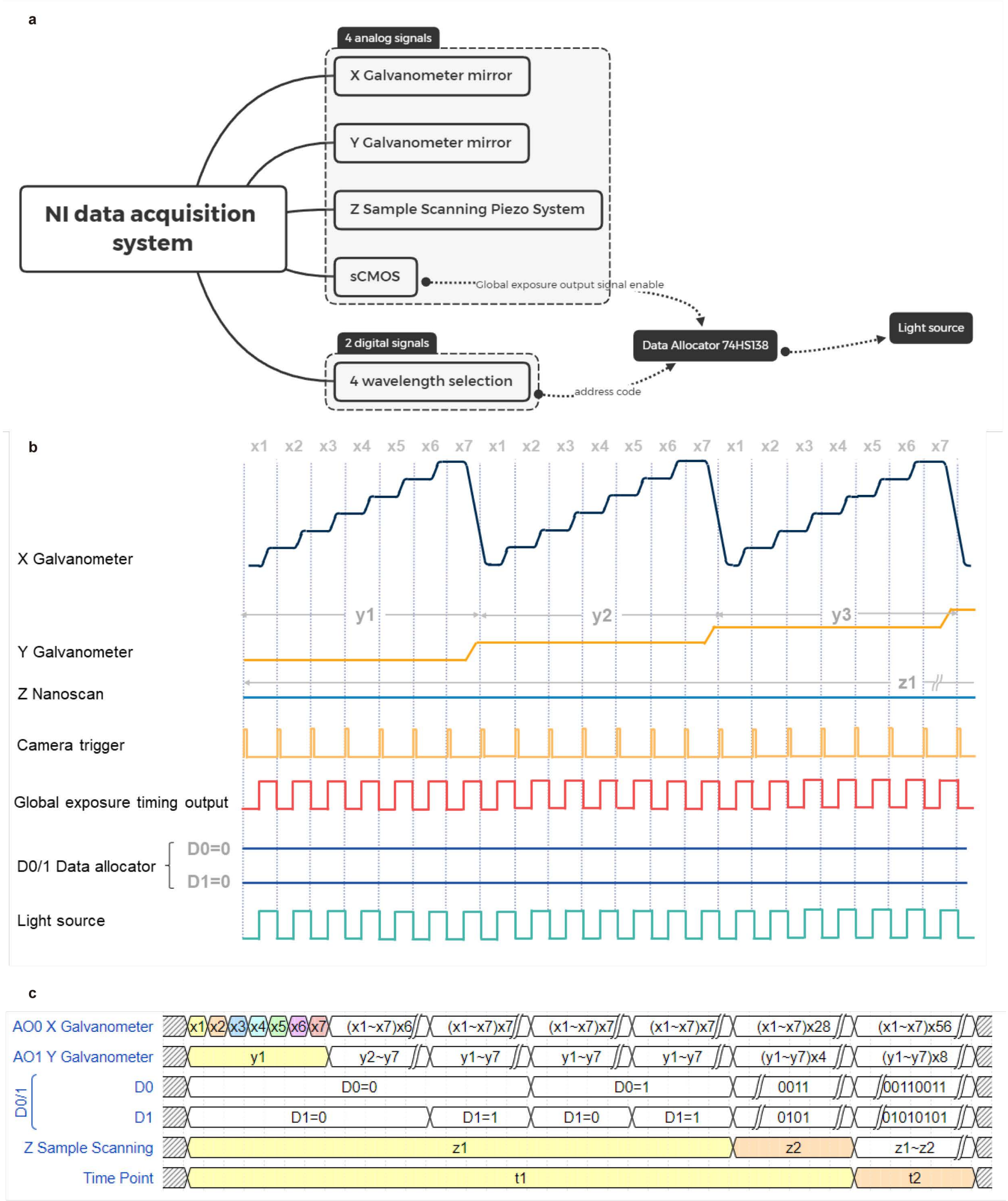

Supplementary Fig. 2. Instrument control and data acquisition process with two-dimensional scanning. **a** Overview of the instrument control. **b** The example of signal control for five-dimensional acquisition (XYCZT), including four colors, two z slices, and two time points. **c** A more detailed signal synchronization process. See Methods "Instrument control" for a detailed description.

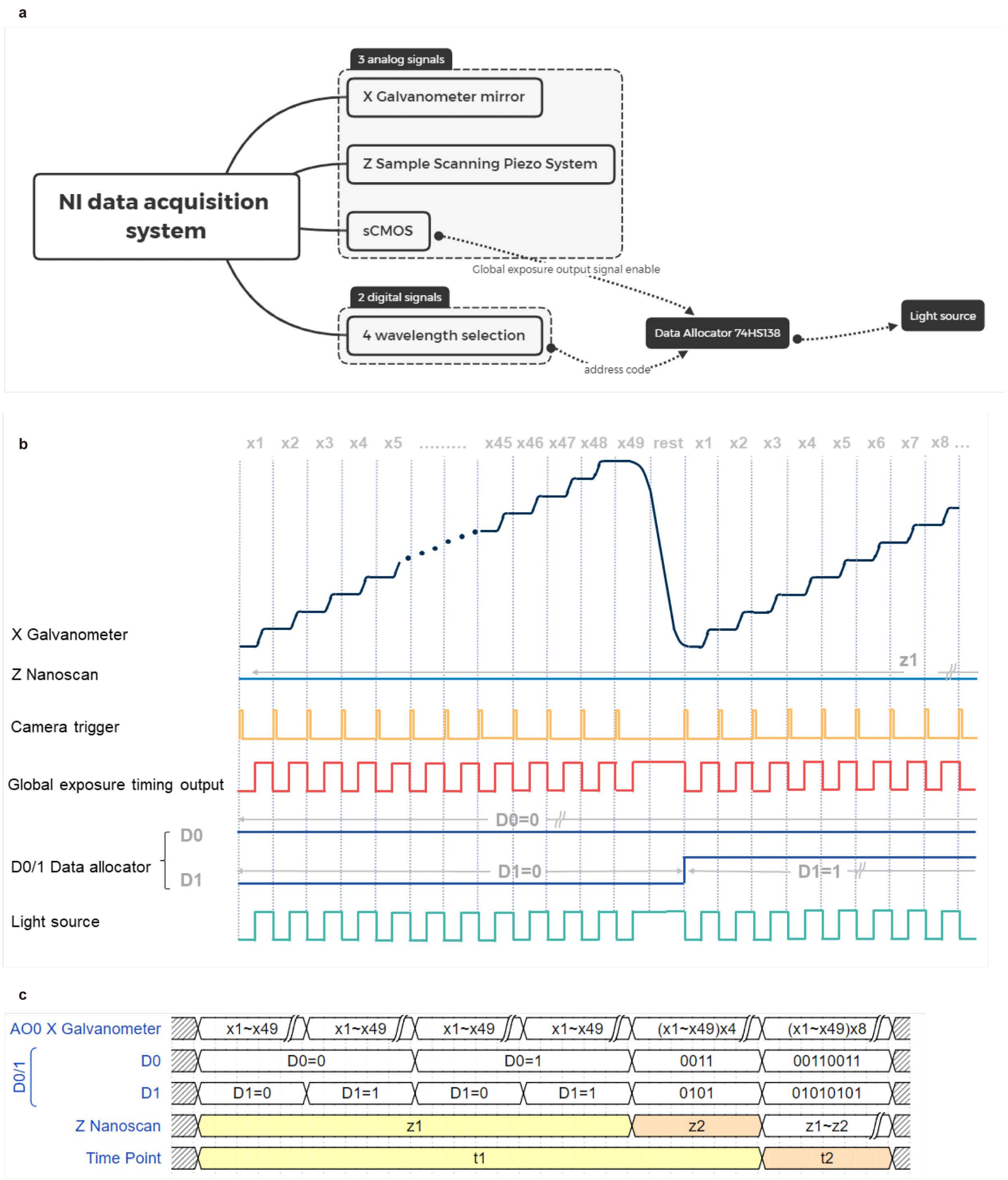

Supplementary Fig. 3. Instrument control and data acquisition process with one-dimensional scanning. **a** Overview of the instrument control. **b** The example of signal control for five-dimensional acquisition (XCZT), including four colors, two z slices, and two time points. Note that the unidirectional scanning of the X Galvanometer acquires information in two dimensions. **c** A more detailed signal synchronization process. See Methods "Instrument control" for a detailed description.

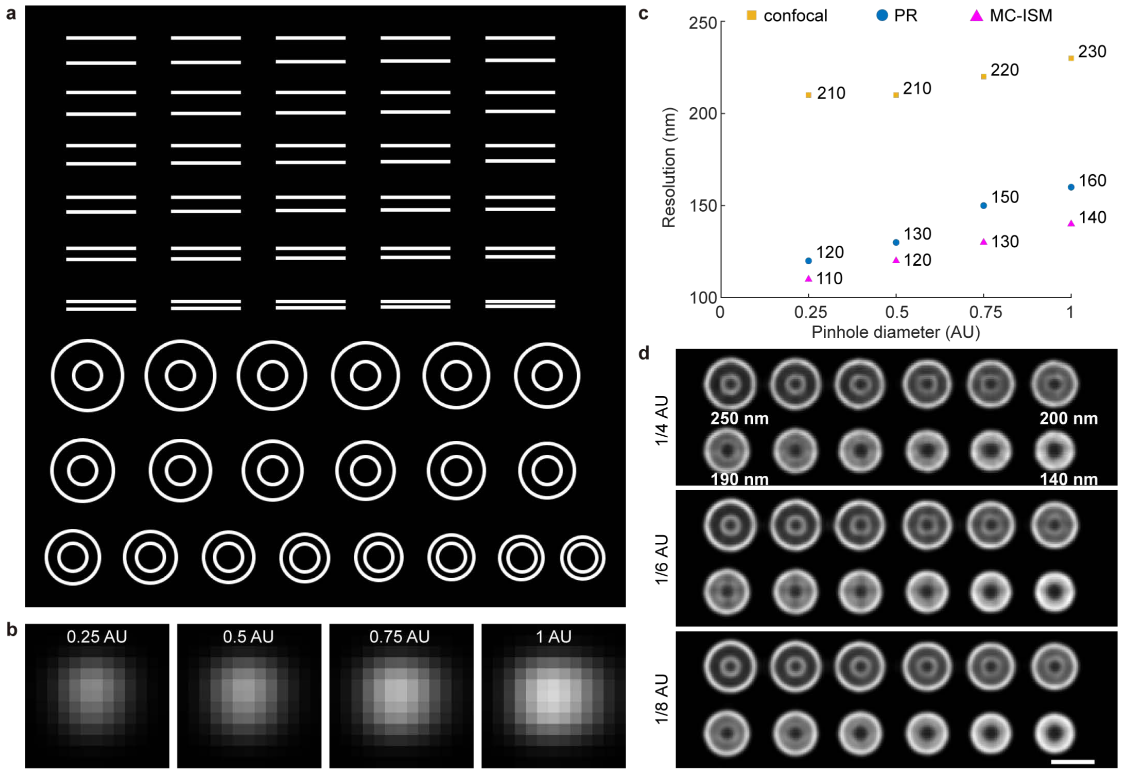

Supplementary Fig. 4. Simulations showing the effect of different excitation pinhole diameters, scan steps, and digital pinhole sizes on MC-ISM imaging results. **a** The ground truth for simulation. **b** Different sizes of excitation pinhole. **c** Under different sizes of excitation pinhole, the minimum resolvable distance of line pairs after reconstruction by Confocal, PR and MC-ISM. **d** Reconstruction results at different scan steps. Scale bar: 1 μm.

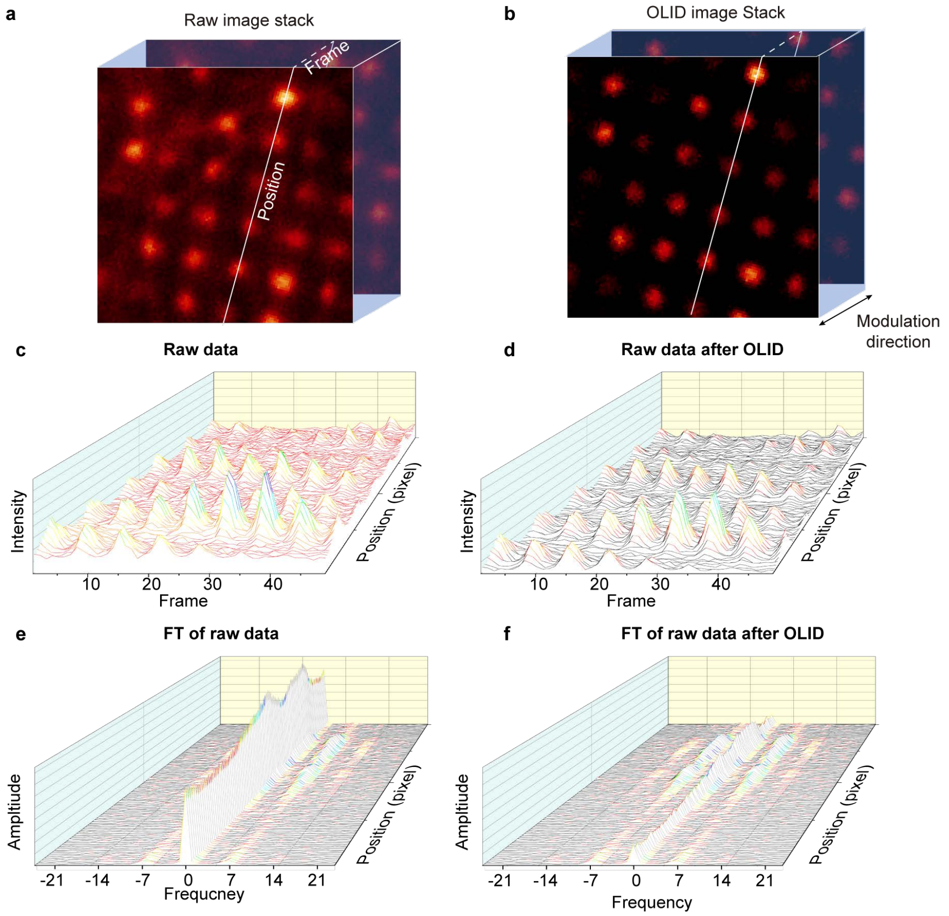

Supplementary Fig. 5. Schematic diagram of the OLID principle. **a** The raw image stack. **b** The image stack after OLID processing. **c**, **d** Waterfall plots generated from **a** and **b** (with the Frame axis corresponding to white dashed lines, the Position axis corresponding to white solid lines, and Intensity values taken from pixel grayscale values). **e**, **f** Fast Fourier transform performed on the data in **c** and **d** along the Frame dimension.

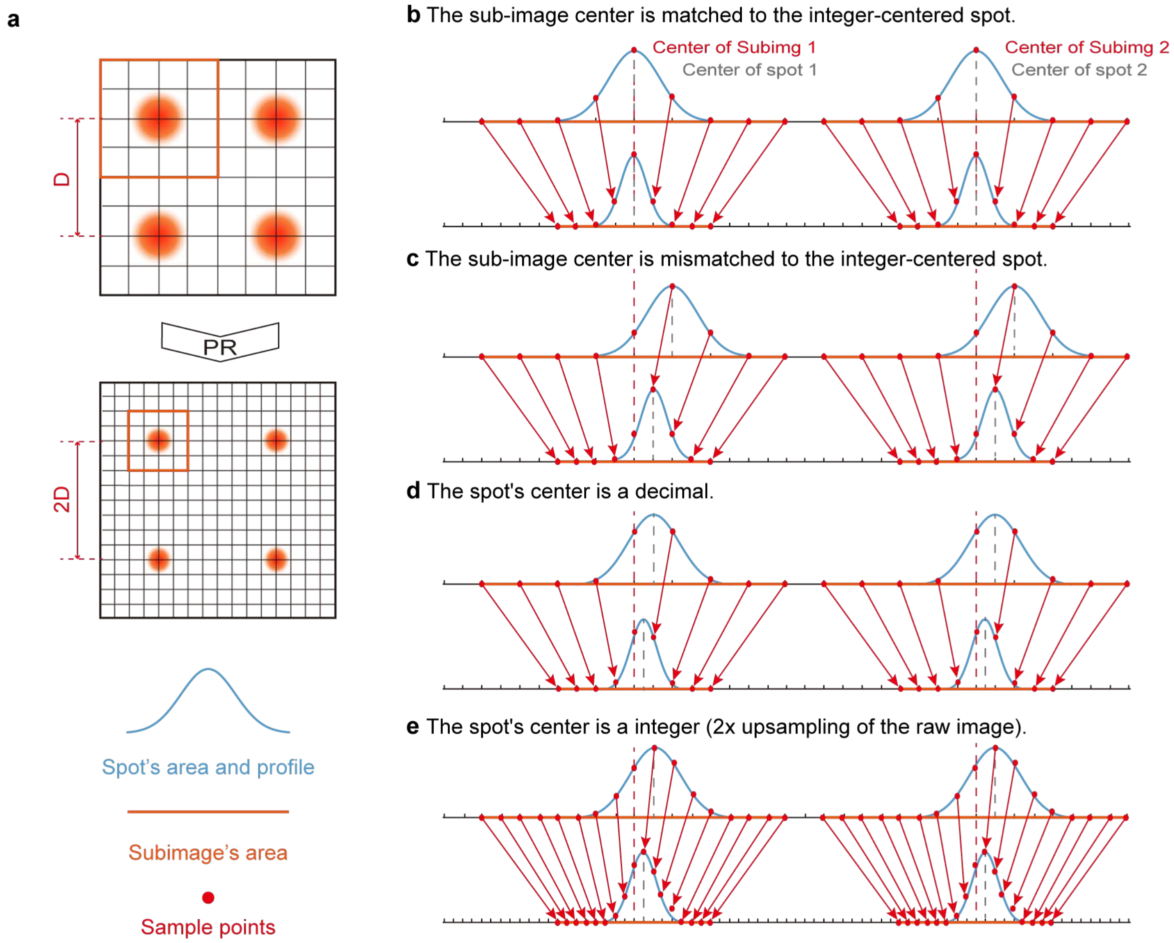

Supplementary Fig. 6. Schematic diagram of PR principle. **a** The MC-ISM reconstruction algorithm achieves PR by doubling the spacing between illumination spots. The sub-image (enclosed in an orange box) is the basic unit for this process, with its center corresponding to the illumination spot's center coordinates obtained through spatial positioning. **b~e** One-dimensional PR schematic diagrams under different positioning conditions. The black coordinate axis represents pixel coordinates, the orange line represents the sub-image range, the blue curve represents the spot intensity distribution (Gaussian profile), the red dot represents pixel sampling of the illumination spot, the red dashed line represents the sub-image center, and the gray dashed line represents the actual center of the illumination spot. **b** When the center coordinates of the illumination spot are integers and align with the sub-image center, PR effectively doubles the distance between illumination spots. **c** When there is a one-pixel offset between the sub-image center and the illumination spot center (both integers), PR is unaffected as long as the sub-image encompasses the entire illumination spot. **d** For non-integer center coordinates of the illumination spot, sub-images can only be

centered on integers, causing pixelation errors. However, PR does not does not change the relative distribution of the actual positions of the illumination spots. **e** Upsampling the raw image aligns non-integer centered illumination spots closer to integer positions, reducing pixelation errors and optimizing PR reconstruction.

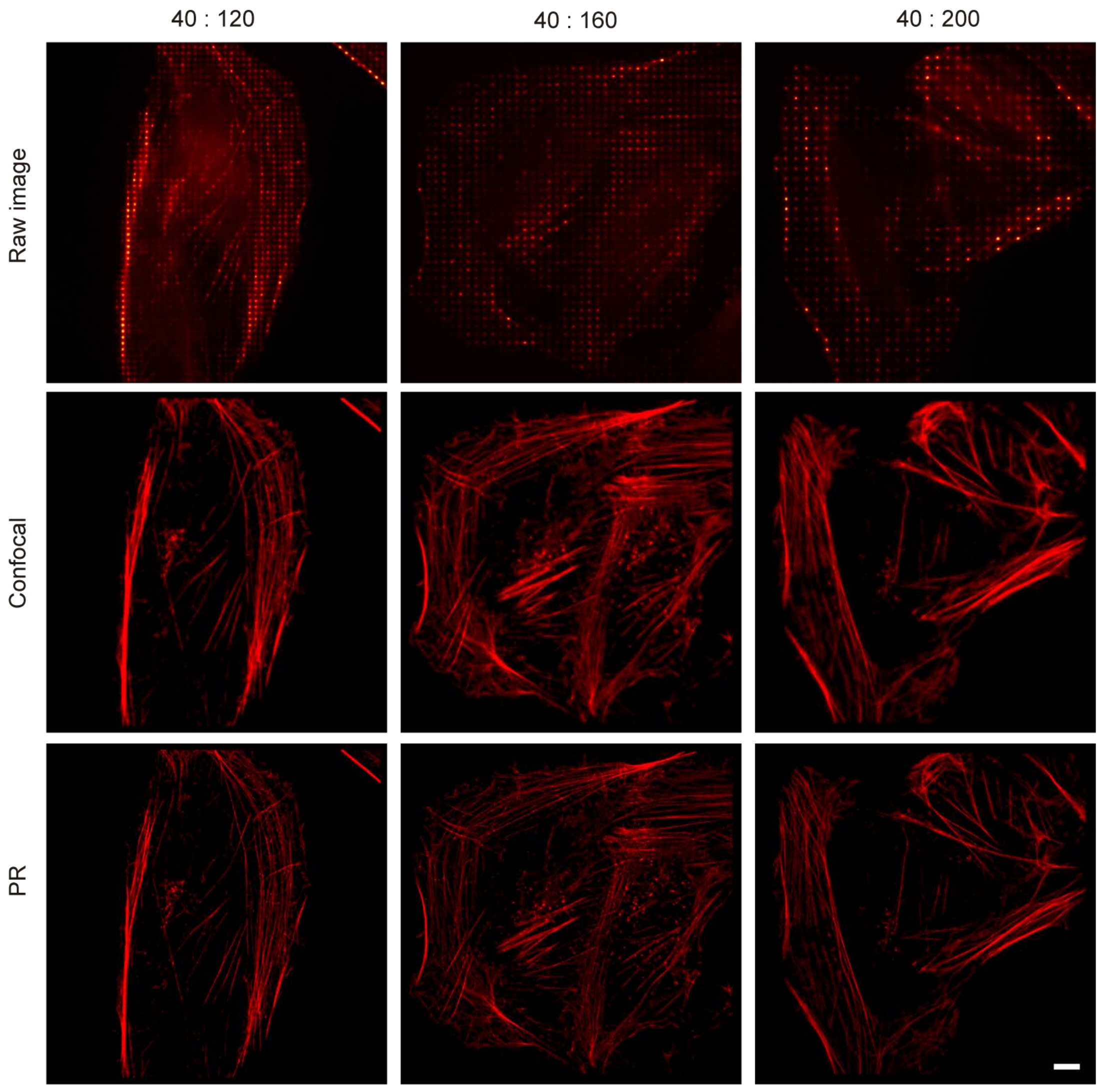

Supplementary Fig. 7. Comparison of MC-ISM imaging results with different pinhole spacing. Phalloidin-Atto 647N labeled actin filaments were imaged by 40 μm:120 μm, 40 μm:160 μm, and 40 μm:200 μm pinhole arrays. Top row: the first frame of raw images. Middle row: results of confocal reconstruction. Bottom row: results of PR reconstruction. As can be seen from the raw image, larger pinhole spacing is more effective in removing out-of-focus signals, which is beneficial to positioning the illumination points accurately. Algorithms that remove out-of-focus signals, such as OLID and digital pinholes, are used for reconstruction, thus there is no obvious difference in image quality caused by background differences from the Confocal and PR reconstruction results. Scale bar: 5 μm.

a

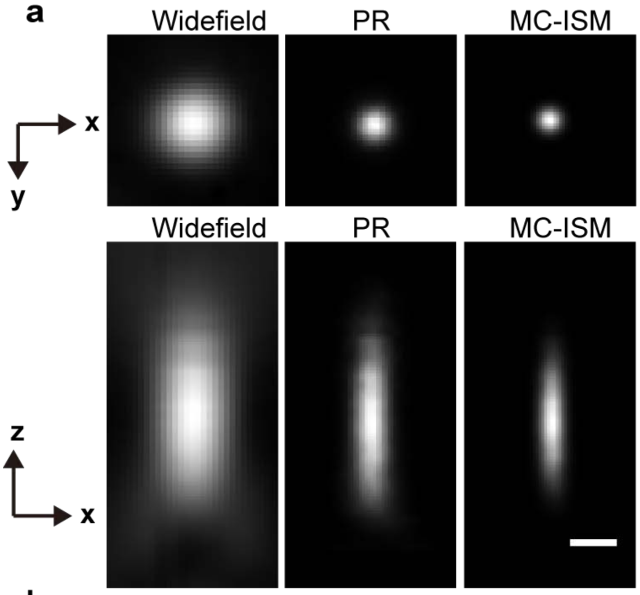

b

	$FWHM_{xy}$	$FWHM_z$
Theory	278 nm	818 nm
Widefield	280 nm	781 nm
PR	170 nm	580 nm
MC-ISM	130 nm	423 nm

Supplementary Fig. 8. a Three-dimensional PSF display of 40 nm beads. Scale bar: 0.5 µm. **b** Measurement results of 40 nm beads by PSFj[2].

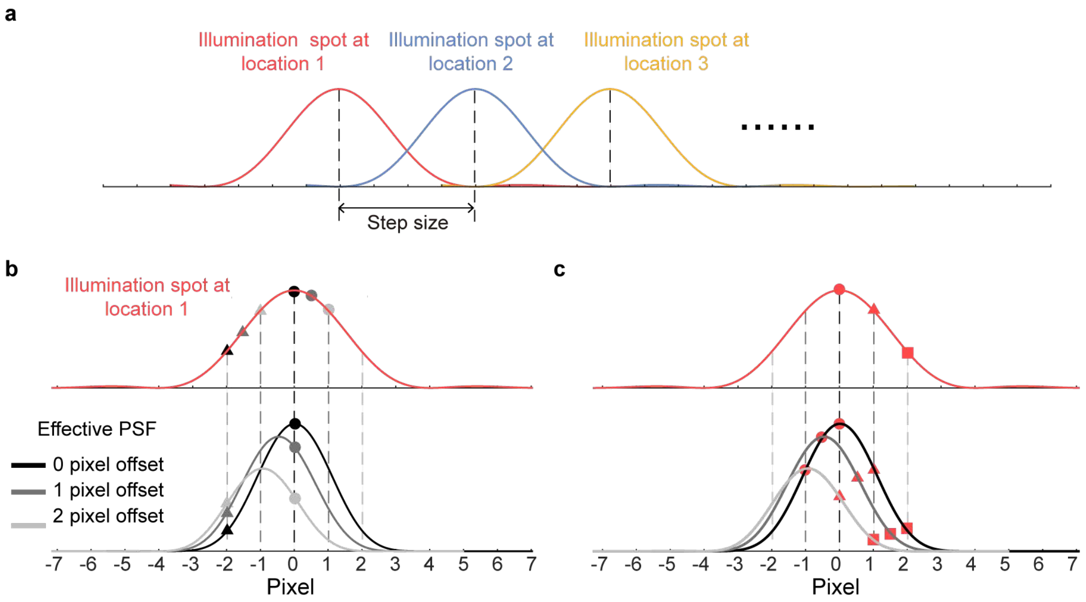

Supplementary Fig. 9. a Frame reduction acquisition schematic shows the coverage area of light spots, indicating an overlap between illumination spots at different scanning locations. **b** The information collected by the same pixel is a weighted sum from different positions of the sample, with the diagram illustrating the composition of information from pixels at the 0 position (circle) and the -2 position (triangle). **c** The same position on the sample can be repeatedly detected by multiple pixels, with the diagram illustrating the detection of information at sample equivalent pixel positions 0 (circle), 1 (triangle), and 2 (square).

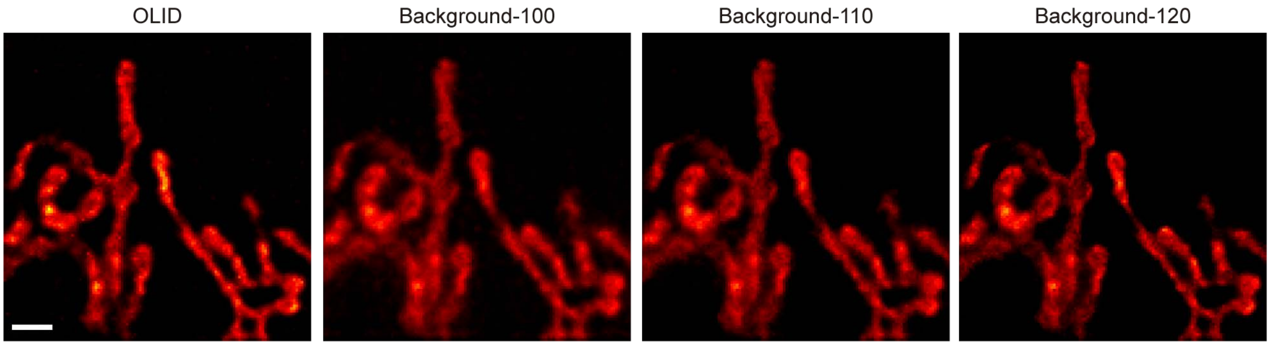

Supplementary Fig. 10. Comparison of the results of OLID preprocessing and the direct background subtraction in the jRL reconstruction. Scale bar: 1 μm.

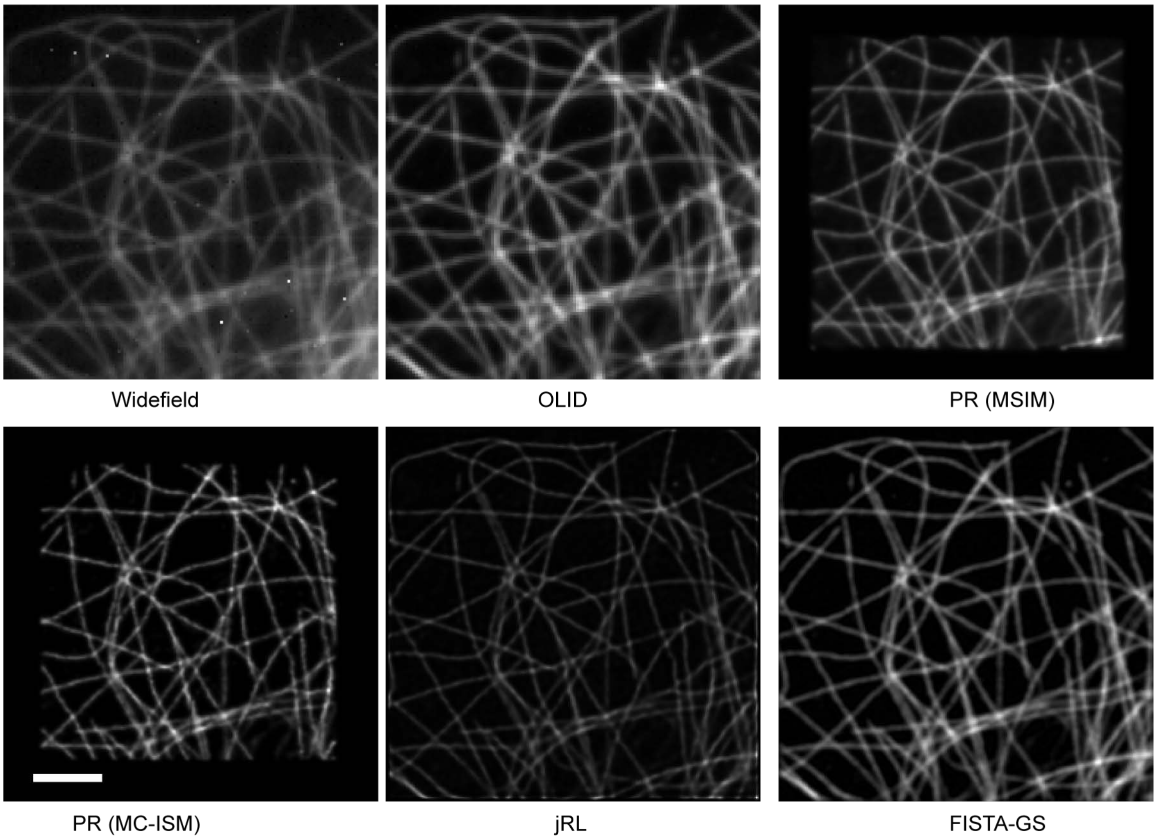

Supplementary Fig. 1 1. Comparative results of different reconstruction algorithms. The source data is derived from the open-source dataset included with MSIM[3]. Scale bar: 2.5 μm.

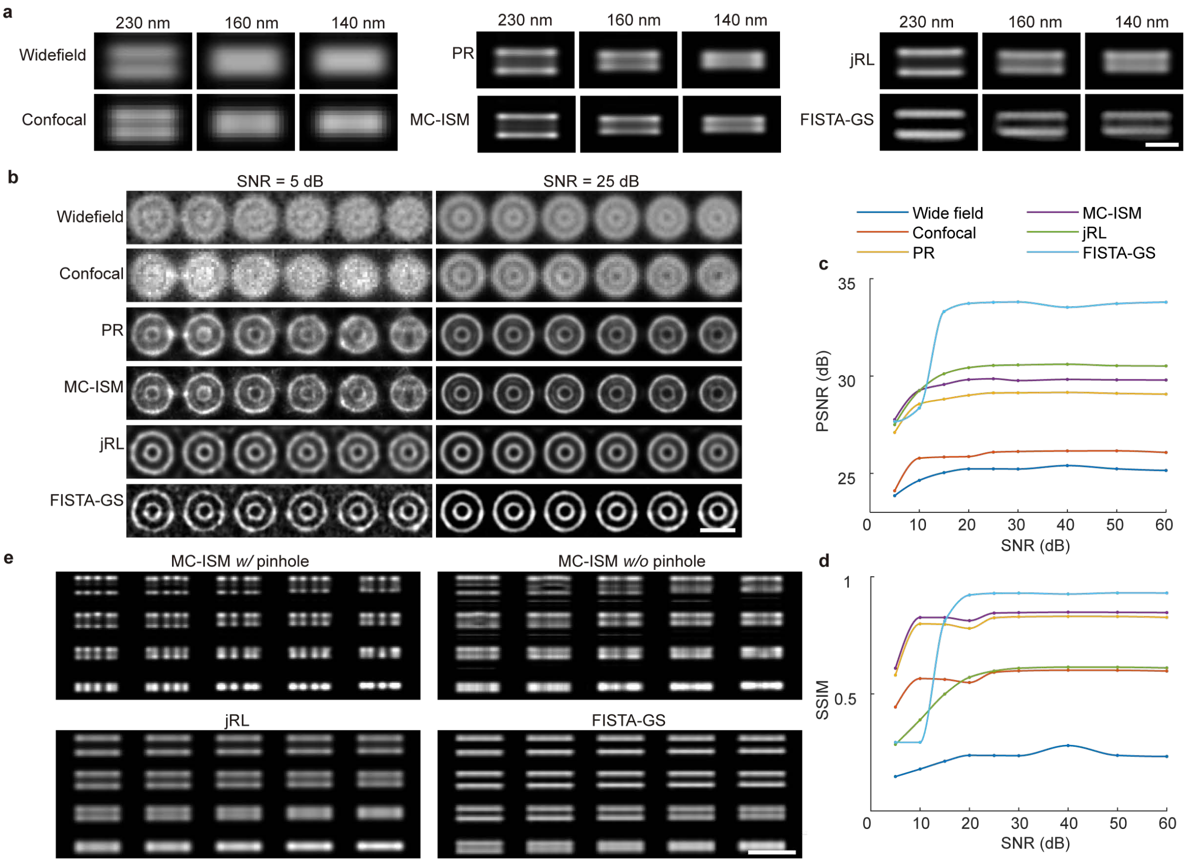

Supplementary Fig. 12. Resolution and image quality assessment for multiple reconstruction methods. **a** Reconstruction results of widefield, confocal, PR, MC-ISM, jRL and FISTA-GS of line pairs at 230 nm, 160 nm and 140 nm distance. Scale bar: 0.5 µm. **b** Reconstruction results at SNR of 5 dB and 25 dB, respectively. Scale bar: 1 µm. **c**, **d** As the SNR of the original image increases, the SSIM (Structural Similarity) and PSNR (Peak Signal-to-noise Ratio) of the reconstructed images using various methods change. **e** Reconstruction results of different methods with a scan step of 0.5 AU. Scale bar: 1 µm.

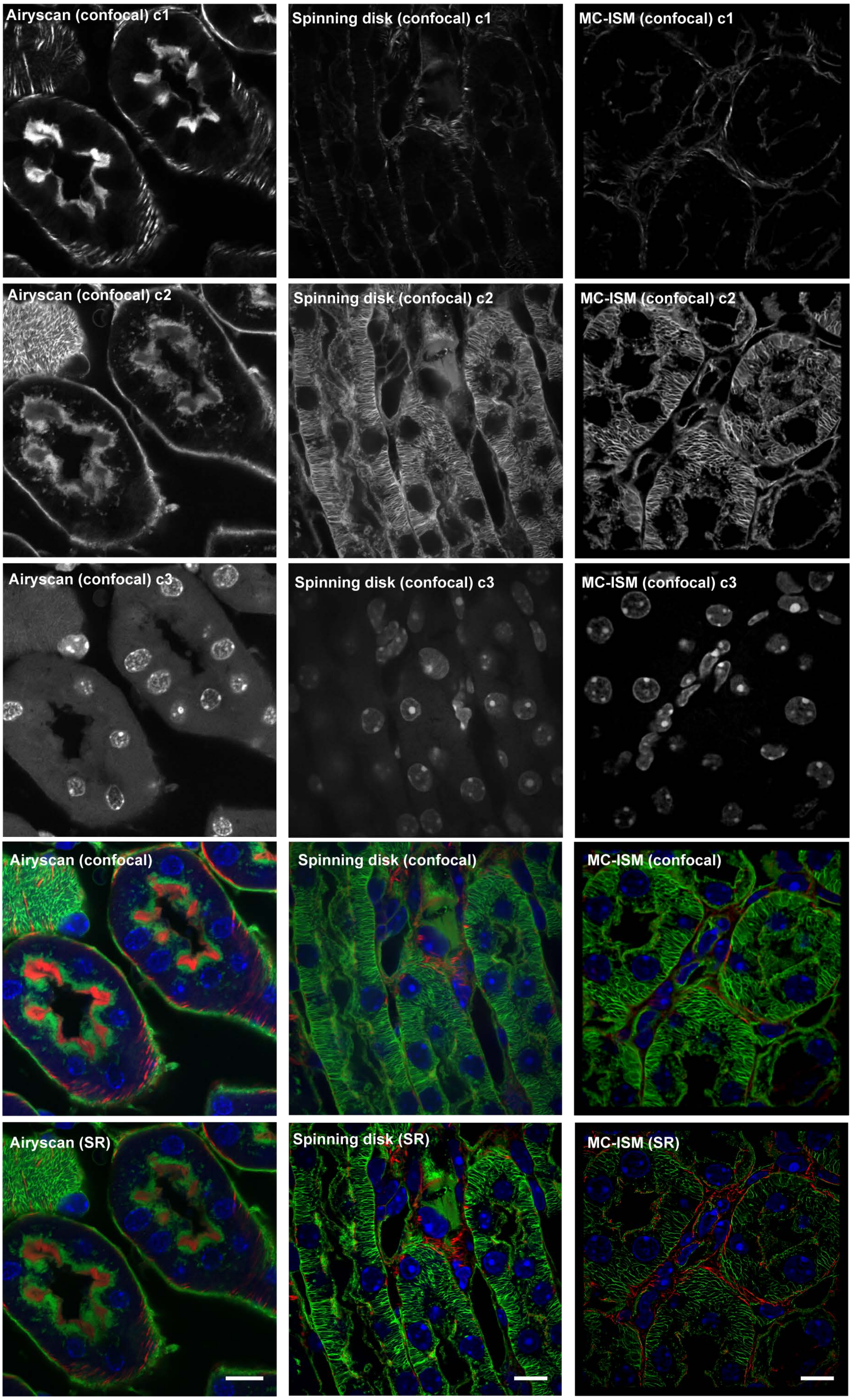
Airyscan (confocal) c1
Spinning disk (confocal) c1
MC-ISM (confocal) c1
Airyscan (confocal) c2
Spinning disk (confocal) c2
MC-ISM (confocal) c2
Airyscan (confocal) c3
Spinning disk (confocal) c3
MC-ISM (confocal) c3
Airyscan (confocal)
Spinning disk (confocal)
MC-ISM (confocal)
Airyscan (SR)
Spinning disk (SR)
MC-ISM (SR)

Supplementary Fig. 13. Comparison of optical sectioning and resolution between MC-ISM, Airyscan and spinning disk in mouse kidney section. The first to third rows are the imaging results of the three channels respectively, the fourth row is the merged result, and the fifth row is the super-resolution result. The Airyscan (Zeiss, Germany) data is processed by Zeiss Zen software. The spinning disk data is obtained through LiveSR spinning disk confocal with a 1.7× magnification module (Gataca systems, France) and deconvolved by Huygens (SVI, Netherlands). Scale bars in Airyscan, spinning disk, and MC-ISM are 10 μm, 16 μm and 8 μm, respectively.

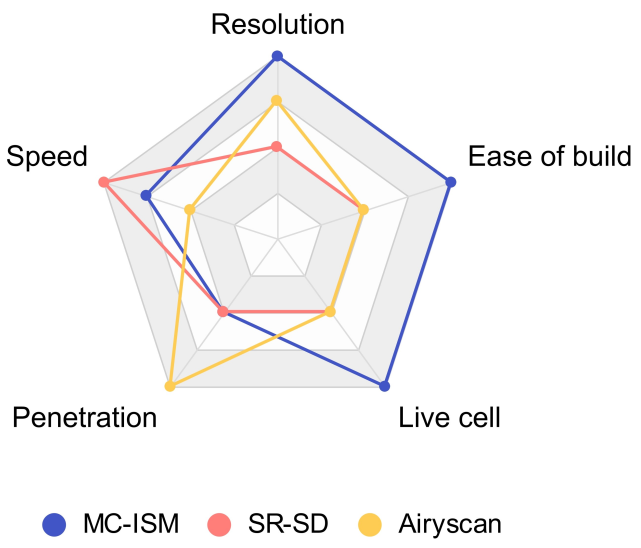

Supplementary Fig. 14. A comparative analysis radar chart of the comprehensive performance of MC-ISM, SR-SD, and Airyscan.

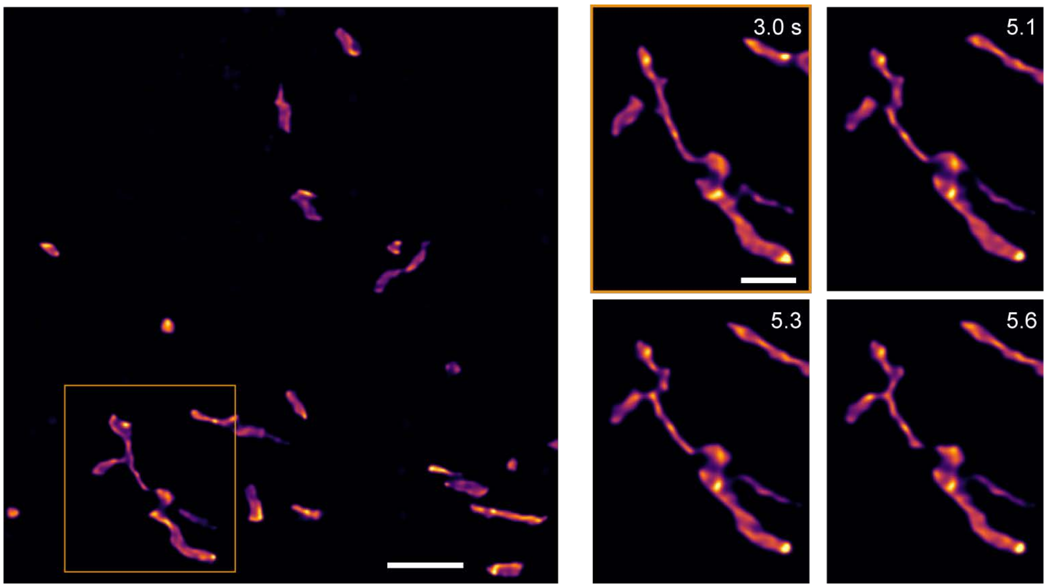

Supplementary Fig. 15. Plant mitochondrial fusion process. The scale bar of the left image is 2.5 μm, and the scale bar of the magnified image on the right is 1 μm.

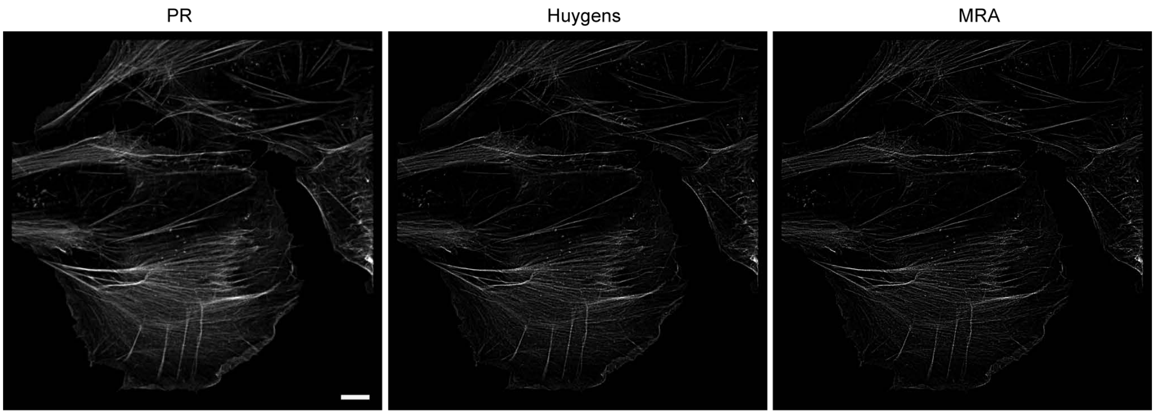

Supplementary Fig. 16. The PR reconstruction results, as well as a comparison of the effect further deconvolved by Huygens (SVI, Netherlands) with default parameters and Multi-Resolution Analysis Deconvolution (MRA). Scale bar 10 um.

Table. S1 List of components used in the MC-ISM.

Component	Manufacturer	Notes	Link	Replaceable	Quantity	Price (RMB)
ACL2520U-A	Thorlabs	Collimating lens	https://www.thorlabs.com/newgrouppage9.cfm?objectgroup_id=3835&pn=ACL2520U#4361	MAC4606-A (LBTEK)	1	295
CFS1/M	Thorlabs	For mounting and manually switching pinhole arrays.	https://www.thorlabs.com/newgrouppage9.cfm?objectgroup_ID=10262	ELL9K (Automatic switching)	1	1,585
Pinhole Array	Juzhike optoelectronics	For producing multi-focal points	http://www.jzkgd.com/		1	1,000
AC254-075-A-ML	Thorlabs	Relay lenses (scan lens) 1 and 2	https://www.thorlabs.com/newgrouppage9.cfm?objectgroup_id=2696		2	1,015
KC1XY/M	Thorlabs	Lens Mount (cage-compatible)	https://www.thorlabs.com/newgrouppage9.cfm?objectgroup_id=185	CXY1A (Thorlabs)	3	1,678
S-8107	Sunny Technology	For 1D small angle scanning	http://www.sunny-technology.com/pro_con.aspx?type=25&id=794	GVS202+ GPS011-EC (Thorlabs)	1	5,000
GCM001	Thorlabs	Galvo Mount (cage-compatible)	https://www.thorlabs.com/newgrouppage9.cfm?objectgroup_id=3770	GCM102/M (Thorlabs)	1	1,442
ER3-P4	Thorlabs	Cage Assembly Rod	https://www.thorlabs.com/newgrouppage9.cfm?objectgroup_ID=4125		2	236
89402 Multi LED set	Chroma	Multi-band filter set (Ex, DM, Em)	https://www.chroma.com/products/set/89402-et-391-32-479-33-554-24-638-31-multi-led-set		1	11,160
#58-874	Edmund optics	Kinematic dichroic mirror mount	https://www.edmundoptics.cn/f/kinematic-filter-mounts/13406/		1	1,040
105mm F2.8 DG DN MACRO | Art	SIGMA	single lens reflex (SLR) lens	https://www.sigma-global.com/en/lenses/a020_105_28/		1	2,800
						Total: 31,858

Table. S2 Comparison of different reconstruction methods for noise-free images

	SSIM	PSNR	Resolution (nm)
Wide field	0.64	25.95	230
Confocal	0.70	26.94	230
PR	0.86	29.46	160
MC-ISM	0.84	29.82	140
jRL	0.88	30.87	140
FISTA-GS	0.92	32.39	110

Table. S3 Comparison of Airyscan, SR-SD and MC-ISM implementation methods.

	**Airyscan**	**SR-SD**	**MC-ISM**
**Light source**	Single mode laser	Single mode laser	LED / Multimode Laser / Single mode laser
**Detector**	a hexagonally packed detector (PMT, QE:45%) array	sCMOS (QE: 82%)	sCMOS (QE: 82%)
**Scanning component**	Single point raster scanning	Multi-point Archimedean spiral scanning	Multi-point single-axis linear scanning
**Algorithm**	Pixel reassignment	Optical pixel reassignment	Pixel reassignment / Multi-image deconvolution frame reduction algorithm

Table. S4 Comparison of related technical performance based on ISM.[a]

Technology	Lateral Res. (nm)	Axial Res. (nm)	Speed	FOV (μm)	Multi-color	Live cell[b]	Ease of build[c]	Costs[d]	Rec. method	Refs.
**MC-ISM**	130	330	16.7 fps with 33 μm ×17 μm	100 × 100	3	★★★	★★★	$	25 raw data	
**MSIM**	145	400	1fps with 48 μm × 49 μm	48 × 49	2	★★	★★★	$$	224 raw data	[3]
**RCM**	170 $(\sqrt{2}\times)$		1fps with 11 μm × 11 μm		1	★	★	$$	Optical	[4]
**OPRA**	327 (63×/0.7 NA oil)				1	★	★	$$	Optical	[5]
**iSIM**	145	350	100 fps with 68μm × 45 μm	68 × 45	2	★★	★	$$	Optical	[6]
**CSD-ISM**	130		1 fps with 5.28 μm× 5.28 μm	34 × 34	3	★★	★★	$$$	250 raw data	[7]
**SD-OPR**	106	268		58 × 50	1	★★	★★	$$$	Optical	[8]
**SPAD-ISM**	193 $(\sqrt{2}\times)$				2	★★★	★★	$$	Single point scan	[9]

[a]All the data mentioned above are sourced from research papers.

[b]The more ★, the more friendly for live cell imaging.

[c]The more ★, the simpler the system is to build.

[d]The more $, the higher the system cost.

Supplementary Note 1. Comparison of 1D and 2D scanning

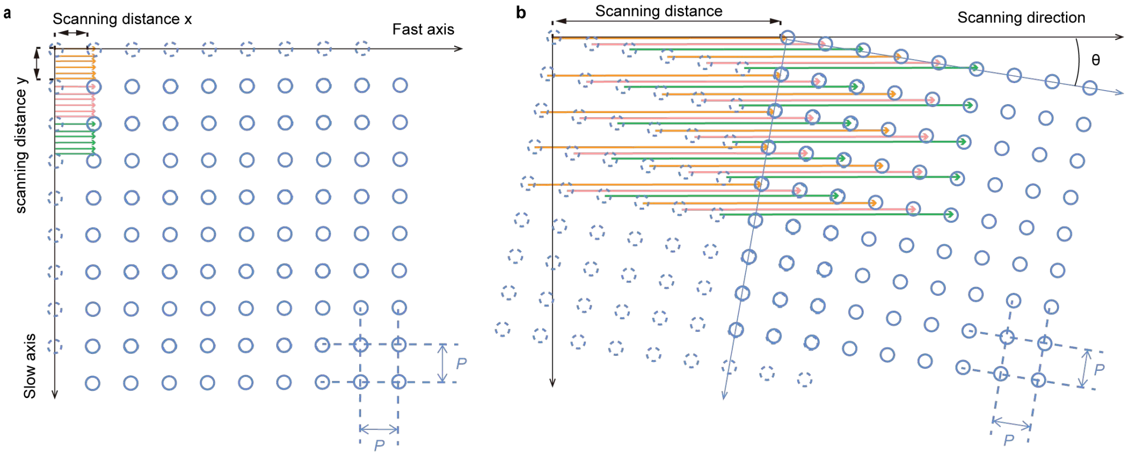

Supplementary Fig. 17 Schematic diagram of one-dimensional scanning (right) and two-dimensional (left) scanning

The configuration for two-dimensional scanning consists of two orthogonal galvanometers completing scanning along the fast axis (x galvanometer) and slow axis (y galvanometer) directions, respectively (Supplementary Fig. 17a). The pinhole array arrangement direction is consistent with the scanning direction. Due to the different motion properties of galvanometers forward and backward sweep, we use unidirectional scanning, that is, each time the x galvanometer completes scanning along the fast axis direction, it will reset to the starting point, then the y galvanometer steps forward once, and the x galvanometer scans again along the fast axis direction, and so on. Assuming that the pinhole spacing is P and each scanning step is 0.25 AU, to achieve uniform illumination of the entire FOV, the number of steps required along the fast axis and slow axis is

$$n = \frac{P}{0.25\mathrm{AU}} - 1. \tag{S1}$$

Therefore, the number of raw images $N_{2\mathrm{D}}$ is

$$N_{2\mathrm{D}} = n^2. \tag{S2}$$

The configuration for one-dimensional scanning only consists of one galvanometer (Supplementary Fig. 17b). The difference is that the pinhole array is tilted at an angle θ with the original fast axis direction. Scanning is only performed forward along the original fast axis direction, and there is no reset in the fast direction or step in the slow

axis direction. θ and P need to meet certain conditions to achieve uniform illumination of the entire FOV.

$$P\sin\theta = 0.25\mathrm{AU}. \tag{S3}$$

The number of raw images N_{1D} is

$$N_{\mathrm{1D}} = \left(\frac{P\cos\theta}{0.25\mathrm{AU}}\right)^2. \tag{S4}$$

From this, it can be seen that when changing pinhole array with different configurations, or altering the total number of raw images, it is necessary to adjust the tilt angle θ of the pinhole array. Now we discuss the situation where the change in the number of raw images during one-dimensional scanning leads to a change in the scanning step size. Assuming that the number of raw images is adjusted from N_a to N_b, and the scanning step size along the scanning direction changes from Δs_a to Δs_b. Substituting (S3) into (S4) easily yields $N_{\mathrm{1D}} = \cot^2\theta$. Meanwhile, based on the relationship between the total step distance and the number of steps, it is easy to deduce $\Delta s \propto \cos\theta / \sqrt{N_{\mathrm{1D}}}$. Hence,

$$\frac{N_a}{N_b} = \left(\frac{\cot\theta_a}{\cot\theta_b}\right)^2 \Rightarrow \frac{\cot\theta_a}{\cot\theta_b} = \sqrt{\frac{N_a}{N_b}}, \tag{S5}$$

$$\frac{\Delta s_a}{\Delta s_b} = \frac{\cos\theta_a}{\cos\theta_b}\sqrt{\frac{N_b}{N_a}} = \frac{\sqrt{1+\cot^2\theta_b}}{\sqrt{1+\cot^2\theta_a}} = \frac{1}{\sqrt{\frac{N_a}{N_b} - \frac{\frac{N_a}{N_b}-1}{1+\cot^2\theta_b}}}, \tag{S6}$$

When N_a and N_b are respectively 49 and 25, $\Delta s_b = \Delta s_a\sqrt{\frac{N_a}{N_b} - \frac{\frac{N_a}{N_b}-1}{1+\cot^2\theta_b}}$. Δs_b is a variable that varies between Δs_a and $1.4\Delta s_a$ depending on the value of θ_b, and the larger the value of $\theta_b \in \left(0, \frac{\pi}{2}\right)$, the smaller the value of Δs_b.

Supplementary Note 2. Simulations showing the effect of different excitation pinhole diameters, scan steps, and digital pinhole sizes on MC-ISM results

Comprehensive simulations are conducted to explore the relationship between reconstruction performance and reconstruction parameters such as excitation pinhole diameters, scan steps, and digital pinhole sizes. The ground truth image for simulation is illustrated in Supplementary Fig. 4a, which consists of line pairs with a 50 nm line width, and the intervals span from 20 nm to 310 nm in 10 nm increments. Additionally, we utilize a ring-shaped sample with a line width of 50 nm to assess the algorithm's isotropic. The distance between the edges of the concentric circles varies from 50 nm to 250 nm in 10 nm increments. The parameters employed in the simulation process are as follows: the excitation wavelength is 561 nm, the emission wavelength is 580 nm, the NA of the objective lens is 1.49, the original image's pixel size is 10 nm, the system magnification is 100×, and the camera's physical pixel size is 6.5 μm.

The relationship between excitation pinhole diameters and resolution

The excitation pinhole diameters are 0.25 AU, 0.5 AU, 0.75 AU, and 1 AU, respectively (Supplementary Fig. 4b). In experiment excitation pinhole size can be adjusted by employing pinhole array with different pinhole size. It can be found that the number of photons in the excitation spot becomes lower, but the reconstruction resolution increases as the excitation pinhole becomes smaller, Confocal and PR resolutions reach 210 nm and 120 nm, respectively (Supplementary Fig. 4c). Furthermore, the MC-ISM reconstruction result is obtained by applying RL deconvolution on the PR image, and shows nearly twice the resolution improvement compared to the confocal image. However, when the excitation pinhole diameters are already very small, the resolution improvement from deconvolution becomes limited. The reason for this limitation is that the small excitation pinhole has already significantly enhanced the resolution, leaving little room for further enhancement through deconvolution.

Relationship between scan step and resolution

A suitable scanning step distance also plays a key role in the imaging resolution. Too small scan step does not improve resolution but prolongs imaging time or increases phototoxicity. Conversely, too large step introduces artifacts and reduces the resolution of reconstruction results. Hence, striking a balance between imaging speed and reconstruction quality is important. In our simulations, the scan step was set to 1/4 AU, 1/6 AU, and 1/8 AU, while the excitation pinhole size remained unchanged at 1 AU. It becomes apparent that a smaller scan step does not yield higher resolution but results

in more isotropic reconstructions with fewer artifacts (Supplementary Fig. 4d). When the scan step exceeds 0.25 AU, there will be gaps in the reconstruction results (Supplementary Fig. 12e).

Supplementary Note 3. MC-ISM reconstruction in the spatial domain

The image reconstruction process of this method involves five steps: (1) optical lock-in detection (OLID), (2) pixel-level center localization, (3) extraction of sub-images, (4) application of digital pinholes, and (5) pixel reassignment and deconvolution. The reconstruction process is illustrated in Fig. 2a.

(1) Optical lock-in detection

The classic OLID is designed to extract weak signals from noise, whereas OLID in MC-ISM is primarily used for removing out-of focus signal[10]. As shown in Supplementary Figure 5, FFT is conducted on raw images, pixel by pixel, in the modulation direction. The zero-frequency component along the modulation direction is attenuated to eliminate the out-of-focus signal because the zero-frequency component contains all the direct current and alternating current components. The Fourier spectral reveals that, in addition to the 0-frequency component peaking, spikes can also be observed at the frequency of ± 7 and ± 14. The reason is that this is a set of 1D scanning data with 49 steps, the projected position of the scanning spot remains unchanged every 7 frames in the scanning direction, with only a net displacement in the direction perpendicular to the scanning direction. If the pixel is background, a distinct spike at frequency ± 7 will not be displayed, which also confirms that the out-of-focus signal exhibits DC characteristics, while the in-focus signal, being modulated, displays AC characteristics. In MC-ISM, the scanning does not follow a cosine or sine form but is stepwise, which introduces multiple components and prevents the direct extraction of specific components corresponding to the step size. Consequently, we then employ a Gaussian window to suppress high-frequency components, thereby completing the OLID preprocessing.

(2) Determine the pixel-level center position

1） Given that a significant portion of the out-of-focus information is removed by the OLID, it is not prone to obstruct the center positioning. However, preprocessing is necessary if OLID is not performed. Raw images undergo preprocessing involving high-pass filtering, morphological operations to eliminate outliers, and denoising.

2） Then median filter is used to remove isolated points in the image and the Gaussian filter is used to make the gray values distribution of the illumination spots in the image tend to the Gaussian profile.

3） Following the eight-neighborhood rule, the pixel-level center pixels are identified. Set the background threshold, pixels whose gray value is higher than the threshold

are judged as signal and retained, while those lower than the threshold are judged as background and removed.

(3) Extract sub-images

Sub-images are extracted either from raw images or from images post-OLID, based on the position of the pixel-level center point. To ensure complete information, the sub-image size is set to be at least three times the full width at half maximum (FWHM) of the excitation point. Additionally, note that the extracted sub-images should not originate from other preprocessing operations to avoid inducing edge enhancements.

(4) Apply digital pinholes

1） Eliminate parts of the sub-image that are below 30% of the highest gray value to further reduce stray light distributed around the edges.

2） To obtain higher positioning accuracy for accurately adding digital pinholes, it is crucial to accurately determine the subpixel center. First, a Gaussian filter is applied to the image then a quadratic function fit is used to determine the subpixel centers.

3） $Subimg_{pinhole}$ is obtained by adding a Gaussian digital pinhole centered on the sub-pixel center.

4） The confocal image is generated by superimposing the $Subimg_{pinhole}$ according to the positioning center directly.

(5) Pixel reassignment and deconvolution

1) Generating an PR image requires reassigning the positions of the $Subimg_{pinhole}$. First, the pixel coordinate of the original image is interpolated to generate non-integer indexes. The number of indexes of the newly generated coordinate system is twice the original one. We call the pixel coordinate of the PR image and $Subimg_{pinhole}$ $Coord_{PR}$ and $Coord_{Sub,old}$, respectively.
2) According to the sub-pixel center coordinates of the sub-image, the closest index of $Coord_{PR}$ is obtained, which is exactly the position of the sub-image in the $Coord_{PR}$. We call it $Coord_{Sub,new}$. Resampling $Subimg_{pinhole}$ from $Coord_{Sub,old}$ to $Coord_{Sub,new}$, the new sub-image $Subimg_{pinhole,new}$ is obtained.
3) Utilizing the $Coord_{Sub,new}$, and integrateing all $Subimg_{pinhole,new}$ into $Coord_{PR}$, generate the PR image. Subsequently, a deconvolution process is applied to reconstruct the ultimate MC-ISM image.

Supplementary Note 4. The implementation process of jRL and FISTA-GS

The implementation process of jRL

In fluorescence imaging, the camera-collected signal $I(x,y)$ adheres to a Poisson distribution. In wide-field fluorescence imaging, RL deconvolution is commonly employed to enhance resolution. However, in the case of MC-ISM, a super-resolution image is generated from multiple original images acquired through excitation pattern scanning, rendering it amenable to direct reconstruction via jRL deconvolution[11, 12]. In typical ISM setups, the scan step is generally set at a minimum of 1/4 AU, which can result in a relatively high number of raw images, consequently constraining imaging speed. Compared with PR, jRL deconvolution has a theoretical advantage by potentially reducing the required raw images as the scan step increases, thereby improving imaging speed.

According to the Supplementary Note 7, the imaging process of MC-ISM in the spatial domain can be described as follows:

$$I(x,y;x_i,y_i) = I_{\mathrm{IIL}}(x,y;x_i,y_i)\cdot \mathrm{Obj}(x,y) * \mathrm{PSF}_{\mathrm{det}}(x,y). \quad \text{(S7)}$$

For the convenience of subsequent theoretical derivation, we represent (S7) in the form of matrix multiplication:

$$\mathbf{e}_i = \mathbf{H}\mathbf{M}_i\mathbf{o} \quad i = 1,2,\ldots,N_{\mathrm{1D}}. \quad \text{(S8)}$$

Where

$$\begin{aligned} \mathbf{o} &= \mathrm{vec}(\mathrm{Obj}) \\ \mathbf{i}_i &= \mathrm{vec}(I) \\ \mathbf{M} &= \mathrm{Diag}(\mathrm{vec}(I_{\mathrm{ILL}})) \\ \mathbf{H} &= \mathrm{BTTB}(\mathrm{PSF}_{\mathrm{det}}) \end{aligned}. \quad \text{(S9)}$$

The subscript i indicates the index of the image collected during the scanning process. $\mathrm{vec}(\cdot)$ represents the operation of unfolding a two-dimensional matrix into a column vector along the column direction. $\mathrm{Diag}(.)$ denotes a diagonal matrix whose diagonal entries are the entries of the vector inside its brackets. $\mathrm{BTTB}(\mathrm{PSF}_{\mathrm{det}})$ denotes a block Toeplitz matrix composed of $\mathrm{PSF}_{\mathrm{det}}$, where each block is also a Toeplitz block (BTTB is the abbreviation for block Toeplitz with Toeplitz blocks[13]). Through the transformation of (S9), we represent a two-dimensional image using a column vector and use matrix multiplication to represent the illumination pattern

modulation and point spread function convolution. The image we actually detect is a random variable following the Poisson distribution with a mean of $\mathbf{e}_i$. Let the detected image be $\mathbf{m}_i$, then the joint probability P is

$$P(\mathbf{m}_i \mid \mathbf{o}) = \prod_r \frac{\exp(-e_{i,r}) e_{i,r}^{m_{i,r}}}{m_{i,r}!}, \tag{S10}$$

Non-bold e and m represent the elements of the corresponding vectors, and r is the index traversing each element. The product is taken because the value of each pixel is independent of each other. Taking the negative logarithm of (S10) and discarding the constant terms, we obtain the log-likelihood function $L(\mathbf{o})$. The optimal $\mathbf{o}$ minimizes $L(\mathbf{o})$:

$$\mathbf{o} = \arg\min_{\mathbf{o}} L(\mathbf{o}) = \arg\min_{\mathbf{o}} \mathbf{1}^T (\mathbf{e}_i - \mathbf{m}_i \odot \ln \mathbf{e}_i). \tag{S11}$$

Where, $\mathbf{1}$ is an all-one vector, and $\odot$ denotes the Hadamard product. Differentiating the matrix function yields:

$$\mathrm{d}L = \mathrm{tr}\left\{\mathbf{1}^T \mathrm{d}(\mathbf{e}_i - \mathbf{m}_i \odot \ln \mathbf{e}_i)\right\} = \mathrm{tr}\left\{\left(\mathbf{1} - \frac{\mathbf{m}_i}{\mathbf{e}_i}\right)^{\mathrm{T}} \mathbf{M}_i \mathbf{H} \mathrm{d}\mathbf{o}\right\},$$

$$\frac{\partial L}{\partial \mathbf{o}} = \mathbf{M}_i^T \mathbf{H}^T \left(\mathbf{1} - \frac{\mathbf{m}_i}{\mathbf{e}_i}\right). \tag{S12}$$

Where, $\div$ denotes element-wise division, and $\mathrm{tr}\{\cdot\}$ represents the trace operation. For optimization problem (S11), performing gradient descent with the step size t set as $\frac{\hat{\mathbf{o}}_{\text{old}}}{\mathbf{M}_i^T \mathbf{H}^T \mathbf{1}}$:

$$\hat{\mathbf{o}}_{\text{new}} = \hat{\mathbf{o}}_{\text{old}} - t \frac{\partial L}{\partial \mathbf{o}} = \hat{\mathbf{o}}_{\text{old}} \frac{\mathbf{M}_i^T \mathbf{H}_i^T \left(\frac{\mathbf{m}_i}{\mathbf{e}_i}\right)}{\mathbf{M}_i^T \mathbf{H}_i^T \mathbf{1}}. \tag{S13}$$

As MC-ISM involves capturing N_{1D} images, we need to perform a reverse computation based on these N_{1D} images to obtain the optimal $\mathbf{o}$. The imaging model (S8) can be rewritten as a parallelized form:

$$\mathbf{e} = \begin{bmatrix} \mathbf{e}_1 \\ \vdots \\ \mathbf{e}_{N_{\text{1D}}} \end{bmatrix} = \begin{bmatrix} \mathbf{H}\mathbf{M}_1 \\ \vdots \\ \mathbf{H}\mathbf{M}_{N_{\text{1D}}} \end{bmatrix} \mathbf{o}. \tag{S14}$$

Considering that $\mathbf{M}^T \mathbf{H}^T \mathbf{1}$ is a constant scalar, it can be ignored. $\mathbf{M}_i$ is a diagonal

matrix, so its transpose is equal to itself. The transpose of H corresponds to the original convolution kernel $\mathrm{PSF}_{\mathrm{det}}$ rotated by 180°. (S13) can be further simplified to:

$$\hat{\mathbf{o}}_{\mathrm{new}} = \frac{1}{N_{\mathrm{1D}}} \hat{\mathbf{o}}_{\mathrm{old}} \sum_{i=1}^{N_{\mathrm{1D}}} \mathbf{M}_i \mathbf{H}^T \left(\frac{\mathbf{m}_i}{\mathbf{H}\mathbf{M}_i \hat{\mathbf{o}}_{\mathrm{old}}} \right). \tag{S15}$$

This is the jRL algorithm we utilized. According to (S15), the entire process of solving the deconvolution algorithm using jRL is as follow:

Algorithm jRL

Procedure implementation of jRL deconvolution

Input: measured raw images $I_{i,\mathrm{noise}}$ ($i = 1, 2, \ldots, N_{\mathrm{1D}}$), number of iteration N_{iter}, upsampling factor upf, excitation wavelength λ_{ex}, emission wavelength λ_{em}, numerical aperture NA, camera pixel size ps.

Initialize: $k = 0$ $\mathrm{Obj}(x, y) = 1/N_{\mathrm{1D}} \sum_{i=1}^{N_{\mathrm{1D}}} I_{i,\mathrm{noise}}$.

1. Preprocess the raw images: Pad raw images to a square shape (if raw images dimensions are unequal), upsample, and apply OLID.

2. Computer the width of excitation and emission PSF: $\sigma_{\mathrm{ex/em}} = 0.61 \frac{\lambda_{\mathrm{ex/em}}}{\mathrm{NA}} \frac{upf}{2.355 ps}$.

3. Generate the excitation and emission PSF: $\mathrm{PSF}_{\mathrm{ex/em}}(x, y) = \frac{1}{\sigma_{\mathrm{ex/em}} \sqrt{2\pi}} \exp\left(-\frac{1}{2} \frac{x^2 + y^2}{\sigma_{\mathrm{ex/em}}^2} \right)$.

4. Generate the illumination lattic using frequency domain positioning.

5. Generate illumination patterns $I_{\mathrm{IIL}}(x, y; x_i, y_i)$ using (S26) and (S30).

While $k \le N_{\mathrm{iter}}$

6. Do jRL iteration according one time according to (S15)

7. Update $k = k + 1$

End

Output: reconstruction result $\hat{\mathbf{o}}_k$.

The implementation process of FISTA-GS

Unlike jRL, which considers the detected image $I_{i,\text{noise}}(x,y)$ (or the column vector representation $\mathbf{m}_i$) as random variables following a Poisson distribution, we can also view the detected data as random variables perturbed by Gaussian white noise $n(x,y)\sim\mathcal{N}(0,\sigma^2)$:

$$I_{i,\text{noise}}(x,y)=I_i(x,y)+n(x,y). \tag{S16}$$

So $I_{i,\text{noise}}(x,y)\sim\mathcal{N}\left(I_i(x,y),\sigma^2\right)$.We can also derive the joint the log-likelihood function under the normal distribution:

$$L=\frac{1}{2\sigma}\sum_{x,y}\left(I_{i,\text{noise}}(x,y)-I_i(x,y)\right)^2=\frac{1}{2\sigma}\left\|I_{i,\text{noise}}(x,y)-I_i(x,y)\right\|_2^2. \tag{S17}$$

Additionally, constraints can be applied during the reconstruction process. In MC-ISM, the image is reconstructed from multiple frames, and introducing constraints on each frame may lead to potential loss of image information. By adopting a strategy akin to group sparsity[14], FISTA-GS imposes sparsity on the superimposed lattice images, contributing to further enhancement in reconstruction quality. We name this method FISTA-GS.

To simplify the model, the spatial coordinates are expressed as *r*, and the initial position of the scanning galvanometer is directly expressed by the subscript *i*. FISTA-GS takes $O_i(r)$ as the target to be solved instead of $\text{Obj}(r)$. Based on the imaging formation model expressed in (S32), it is known that the ideal captured images $I_i(r)$ can be briefly described as:

$$I_i(r)=I_{\text{IL}i}(r)\cdot \text{O}_i(r)*\text{PSF}_{\text{det}}(r). \tag{S18}$$

Where $\text{PSF}_{\text{det}}(r)$ is the detection PSF, and $I_{\text{IL}i}(r)$ is the illumination pattern.The FISTA-GS is aimed at solving the following optimization problems:

$$\begin{aligned}O_i&=\arg\min_{O_i}\frac{1}{2}\sum_i\left\|I_i(r)-I_{i,\text{noise}}(r)\right\|_2^2+\lambda\left\|\sum_i O_i(r)\right\|_1\\&=\arg\min_{O_i}\left[\frac{1}{2}\sum_i\left\|\left(I_{\text{IL}i}(r)\cdot O_i(r)\right)*PSF_{\text{det}}(r)-I_{i,\text{noise}}(r)\right\|_2^2+\lambda\left\|\sum_i O_i(r)\right\|_1\right].\\&=\arg\min_{O_i}\left[f(\mathbf{o})+\lambda g(\mathbf{o})\right]\end{aligned} \tag{S19}$$

Where, $\|\cdot\|_1$ and $\|\cdot\|_2$ denote L1 and L2 norm respectively. Let $F(\mathbf{o})=f(\mathbf{o})+\lambda g(\mathbf{o})$, $f(\mathbf{o})$ is a differentiable with Lipschitz continuous gradient L, and $g(\mathbf{o})$ is closed and convex. Considering the quadratic approximation of $F(x)$ at a given point $\mathbf{y}$:

$$Q_L(\mathbf{o},\mathbf{y})=f(\mathbf{y})+\left\langle\mathbf{o}-\mathbf{y},\nabla f(\mathbf{y})\right\rangle+\frac{L}{2}\|\mathbf{o}-\mathbf{y}\|^2+\lambda g(\mathbf{o}), \tag{S20}$$

a unique minimization operator $p_L(\mathbf{x})$ can be defined as:

$$p_L(\mathbf{y}) = \arg\min_{\mathbf{o}} Q_L(\mathbf{o},\mathbf{y}) = \arg\min_{\mathbf{o}} \left\{ \lambda g(\mathbf{o}) + \frac{L}{2} \left\| \mathbf{o} - (\mathbf{y} - \frac{1}{L}\nabla f(\mathbf{y})) \right\|_2^2 \right\}. \quad \text{(S21)}$$

With the help of proximal gradient descent, the proximal gradient operator $p_L(y)$ can be expressed as follows:

$$\begin{aligned} p_L(y) &= \arg\min_{O} Q_L(O,y) \\ &= \arg\min_{O} \left[\frac{L}{2} \left\| O - (y - \frac{1}{L}\nabla f(y)) \right\|_2^2 + \lambda g(O) \right] \end{aligned}. \quad \text{(S22)}$$

Similar to the soft-threshold operation, in the region $\sum_i O_i(r) > 0$, the derivative can be obtained $O = y - \frac{1}{L}\nabla f(y) - \frac{\lambda}{L}$; in the region $\sum_i O_i(r) > 0$, the derivative can be obtained $O = y - \frac{1}{L}\nabla f(y)$; in the region $\sum_i O_i(r) > 0$, the derivative can be obtained $O = y - \frac{1}{L}\nabla f(y) + \frac{\lambda}{L}$. Therefore, the key to the problem is how to find the gradient of $f(y_i)$. When solving $\nabla f(y_i)$, according to the chain rule, the problem can be transformed into $\frac{\delta f}{\delta O_i(r)} = \frac{\delta f}{\delta I_i}\frac{\delta I_i}{\delta O_i}$. In order to explain the convolution, this article defines the coordinates of I_i as r', consider one point r_0 of O_i, we can get:

$$I_i(r') = \left[I_{\mathrm{IIL}i}(r_0) \cdot O_i(r_0) \right] \cdot \mathrm{PSF}_{\mathrm{det}}(r' - r_0). \quad \text{(S23)}$$

Therefore,

$$\begin{aligned} \delta f &= \int \frac{\delta f}{\delta I_i(r')} \delta I_i(r') \mathrm{d}r' \\ &= \int \left(I_i(r') - I_{i,\mathrm{noise}}(r') \right) \delta I_i(r') \mathrm{d}r' \\ &= I_{\mathrm{IIL}i}(r_0) \cdot \int \left(I_i(r') - I_{i,\mathrm{noise}}(r') \right) \cdot \mathrm{PSF}_{\mathrm{det}}(r' - r_0) \mathrm{d}r' \cdot \delta O_i(r_0) \\ &= I_{\mathrm{IIL}i}(r_0) \cdot \left[(I_i(r) - I_{i,\mathrm{noise}}(r)) * \mathrm{PSF}_{\mathrm{det}}(-r) \right]_{r=r_0} \cdot \delta O_i(r_0) \end{aligned}, \quad \text{(S24)}$$

$$\nabla f = I_{\mathrm{IIL}i}(r) \cdot \left[\left(I_i(r) - I_{i,\mathrm{noise}}(r) \right) * \mathrm{PSF}_{\mathrm{det}}(-r) \right]. \quad \text{(S25)}$$

The iterative update process can refer to the FISTA algorithm[15]. The difference is that in the same iteration cycle, the results corresponding to different *i* are gradually updated and the GS constraint is implemented. The precise implementation process is as follows:

Algorithm FISTA-GS

Procedure implementation of FISTA-GS deconvolution

Input: measured raw image $I_{i,\text{noise}}$ ($i = 1, 2, ..., N_{1D}$), image background b^0, number of iterations N_{iter}, Lipschitz constant L, regularization coefficient $\lambda = \{\lambda_1, \lambda_2\} > 0$, upsampling factor upf, excitation wavelength λ_{ex}, emission wavelength λ_{em}, numerical aperture NA, camera pixel size ps.

Initialize: $k = 0$, $O_i^0 = I_{i,\text{noise}}, x_0 := \{O_i^0, b^0\}, y_1 := x_0\ t_1 := 1.0, l_0 = L, \eta = 1.1$,

1~5 are the same as Algorithm jRL.

While $k \le N_{\text{iter}}$

6. **Calculate the forward result according to the formula (S18).**

7. **Calculate the gradient of** f **according to the formula (S21) ~ (S25).**

8. **Find the smallest nonnegative integer** i_k **such that:** $\overline{l} = \eta^{i_k} l_{k-1}$ **and**

$$F\left(p_{\overline{l}}\left(y_k\right)\right) \le Q_{\overline{l}}\left(p_{\overline{l}}\left(y_k\right), y_k\right).$$

9. **Update** l_k, x_k, t_{k+1}, y_{k+1} **and** O_i^k **:** $l_k = \overline{l}$, $x_k = p_{l_k}\left(y_k\right)$,

$$t_{k+1} = \frac{1 + \sqrt{1 + 4t_k^2}}{2}, y_{k+1} = x_k + \frac{t_k - 1}{t_{k+1}}\left(x_k - x_{k+1}\right),\ O_i^k = y_k.$$

End

10. $\text{Obj} = \frac{1}{N_{1D}} \sum_{i=1}^{N_{1D}} O_i^k$

Output: reconstruction result Obj.

Supplementary Note 5. Resolution and image quality assessment for multiple reconstruction methods

(1) Comparison of resolution and reconstruction quality of different reconstruction methods

All of the aforementioned methods are used to reconstruct in the absence of noise and the reconstruction results are compared. The simulation parameters are as follows: an emission wavelength of 580 nm, an excitation pinhole diameter of 1 AU, a scan step size of 0.25 AU. The theoretical lateral resolution in widefield imaging of 237 nm. As Supplementary Fig. 12a shows, the resolution of the confocal reconstruction is approximately 230 nm. The resolution of PR reconstruction with digital pinholes is significantly enhanced to approximately 160 nm, achieving an improvement of about √2 times. Subsequent deconvolution further improves the resolution to approximately 140 nm.

(2) Robustness of different reconstruction methods to noise

Upon introducing noise into the original image and applying various reconstruction methods, we observe that the image resolution remained unaltered, but noticeable image artifacts emerged (Supplementary Fig. 12b). The addition of noise introduces localized maximum grayscale pixels in the background regions, a phenomenon that may persist despite preprocessing operations conducted before sub-image extraction. This interference can disrupt the positioning of the illumination point during the PR reconstruction process. Consequently, as the SNR decreases, it becomes essential to appropriately raise the intensity threshold for distinguishing signal from background to uphold reconstruction quality. For both confocal and PR, which selectively read regions with a certain intensity, the reconstruction quality remains unchanged as long as the coordinates of the illumination point are unaffected. We found that when the acquired image quality reaches around 25 dB, the reconstruction quality of different methods tends to stabilize. At this juncture, FISTA-GS exhibits the highest Peak Signal-to-noise Ratio (PSNR) and Structural Similarity (SSIM) values among all methods (Supplementary Fig. 12c and Supplementary Fig. 12d).

(3) The deconvolution method has low requirements on the step size

When the step size is set to 0.5 AU, which is larger than the 0.25 AU, and the standard deviation of the digital pinhole (σ_{pinhole}) is adjusted to σ_{base} , the MC-ISM image exhibits discontinuities (Supplementary Fig. 12e). Upon zooming the digital pinhole to approximately $1.5\sigma_{\text{base}}$, the image structure becomes continuous, although artifacts

remain. Conversely, when the digital pinhole is removed, the MC-ISM image exhibits continuity, yet artifacts are evident. Comparatively, both jRL and FISTA-GS yield superior reconstruction results, with jRL resolving 150 nm line pairs and FISTA-GS resolving 120 nm line pairs. FISTA-GS allows for a relaxation of the scan step to 0.5 AU and a reduction in the number of scan frames without a significant deterioration in resolution.

Supplementary Note 6. Performance comparison of Airyscan, SR-SD and MC-ISM

We have listed the differences in implementation of Airyscan, SR-SD, and MC-ISM in Table. S3, and we also provide a detailed comparison of their performance across the following five aspects:

1. **Resolution**: MC-ISM, Airyscan, and SR-SD are all super-resolution methods based on image-scanning microscopy, theoretically achieving a two-fold resolution enhancement compared to the diffraction limit. Using FRC to measure the results of these three technologies, the resolutions of MC-ISM, SR-SD, and Airyscan are 135 nm, 165 nm, and 146 nm, respectively. Practically, MC-ISM achieves slightly better resolution than both Airyscan and SR-SD. The SR-SD resolution is inferior to algorithmic reconstruction due to optical alignment errors in the detection path and aberrations introduced by the microlenses.
2. **Photobleaching** (**Live Cell Imaging Capabilities**): In Fig. 5a and 5b, we compared photobleaching among MC-ISM, Airyscan, SR-SD, and SIM. Both imaging principles and test results demonstrated that MC-ISM has the lowest photobleaching. The SR-SD system's detection optical path is longer and requires passing through a microlens array, leading to considerable fluorescence decay and pronounced photobleaching or phototoxicity. Since Airyscan is a single-point scanning method, its excitation focus has very high power, and the quantum efficiency (QE) of the PMT array is far inferior to that of sCMOS, resulting in significant photobleaching and phototoxicity.
3. **Imaging Speed**: SR-SD achieves super-resolution imaging through optical pixel reassignment, offering a significant advantage in imaging speed. The multi-point excitation imaging speed of MC-ISM is significantly faster than that of single-point scanning Airyscan.
4. **Penetration Depth**: Since Airyscan uses single-point detection, it has the deepest imaging depth. The MC-ISM technique, using OLID and digital pinholes, has the same out-of-focus signal reduction effect and penetration depth as spinning disk confocal microscopy.
5. **Ease of Build**: The construction cost of MC-ISM is the lowest, using only conventional standard components, all of which can be purchased on the Thorlabs website. In contrast, the array detector used by Airyscan and the dual-microlens array disk used by SR-SD are highly complex proprietary products.

Currently, SR-SD and Airyscan are the most widely used imaging tools in biological research. MC-ISM boasts a slightly superior imaging resolution compared to both systems while maintaining imaging depth comparable to SR-SD. Additionally, it exhibits faster imaging capabilities than Airyscan, though slower than SR-SD. In terms of user-friendliness for live-cell imaging, MC-ISM significantly outperforms both systems. Furthermore, its cost and construction complexity are notably lower than those of the other two technologies. The radar chart comparing the performance of the three technologies is shown in Supplementary Fig. 14. Consequently, the comprehensive performance of MC-ISM positions it as an advantageous tool for in situ imaging of subcellular dynamics within biological tissues. The compatibility of MC-ISM with existing confocal systems and its cost-effectiveness makes it a promising contender to usher in the next era of confocal microscopy.

Supplementary Note 7. MC-ISM imaging formation model

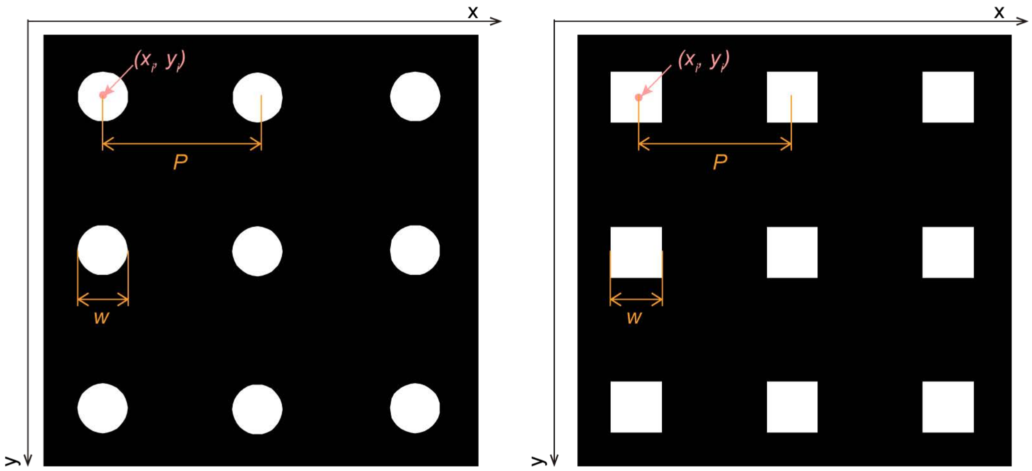

Supplementary Fig. 18 Two-dimensional square (left) and circular (right) pinhole arrays. *P* is the distance between adjacent pinholes, and *w* is the width or diameter of pinholes.

The imaging process of MC-ISM can be modeled in both spatial domain and frequency domain, described as follows.

The laser first passes through a two-dimensional pinhole array, which is expressed as $M(x, y; x_i, y_i)$. To facilitate the derivation of formulas and simplify the expression form, a plane rectangular coordinate system *xoy* is established along the direction of the pinhole arrangement, and square pinholes are used instead of circular pinholes, as shown in Supplementary Fig. 18. The distance between the centers of the two holes is *P*, and the width of the holes is *w*, (x, y) represents the coordinates in the spatial domain, (x_i, y_i) is the relative initial position of the pinhole array illumination modulation for the i_{th} image. Therefore, $M(x, y; x_i, y_i)$ can be expressed as follows:

$$M(x, y; x_i, y_i) = \mathrm{Rect}\left(\frac{x}{w}, \frac{y}{w}\right) * \left[\frac{1}{|P|^2}\mathrm{Comb}(\frac{x - x_0}{P}, \frac{y - y_0}{P})\right]. \qquad \text{(S26)}$$

Where $\mathrm{Rect}\left(\frac{x}{w}, \frac{y}{w}\right)$ is a two-dimensional rectangular function[16] with the width of *w*, and $\mathrm{Comb}\left(\frac{x - x_i}{P}, \frac{y - y_i}{P}\right)$ is a Dirac comb function with *P* as the period and (x_i, y_i) as the center. The initial position of the first image is (x_1, y_1), and (x_i, y_i) increases with each move. Given that the step voltage of the galvanometer is ΔV, the step size of the pinhole is

$$\begin{bmatrix} x_{i+1} - x_i \\ y_{i+1} - y_i \end{bmatrix} = \xi \Delta V \begin{bmatrix} \cos\theta \\ -\sin\theta \end{bmatrix}. \tag{S27}$$

Where ξ is the conversion coefficient from the voltage to the forward step size of the illumination spots on the image surface. From the point spacing uniformity performance shown in Fig. 1e, 1f, it can be considered that different illumination spots in the entire FOV have the same ξ.

If we use the upper sign "~" to mark the Fourier transform of a variable and use (f_x, f_y) as the coordinates in the frequency domain, the Fourier transform of the pinhole array $M(x, y; x_i, y_i)$ can be expressed as follows:

$$\begin{aligned}
\tilde{M}(f_x, f_y; x_i, y_i) &= \frac{1}{|P|^2} \mathcal{F}\left\{ \mathrm{Rect}\left(\frac{x}{w}, \frac{y}{w}\right) \right\} \cdot \mathcal{F}\left\{ \mathrm{Comb}\left(\frac{x - x_i}{P}, \frac{y - y_i}{P}\right) \right\} \\
&= \frac{1}{|P|^2} \mathcal{F}\left\{ \mathrm{Rect}\left(\frac{x}{w}, \frac{y}{w}\right) \right\} \cdot \mathcal{F}\left\{ \sum_{m,n} \delta(x - x_i - mP, y - y_i - nP) \right\} \\
&= \frac{w^2}{|P|^4} \operatorname{sinc}(wf_x) \operatorname{sinc}(wf_y) \cdot \sum_{m,n} \delta\left(f_x - \frac{m}{P}, f_y - \frac{n}{P}\right) \exp\left(-j2\pi f_0 (mx_i + ny_i)\right) \\
&= \sum_{m,n} \frac{w^2}{|P|^4} \operatorname{sinc}\left(\frac{wm}{P}\right) \operatorname{sinc}\left(\frac{wn}{P}\right) \exp\left(-j2\pi f_0 (mx_i + ny_i)\right) \delta\left(f_x - \frac{m}{P}, f_y - \frac{n}{P}\right)
\end{aligned} \tag{S28}$$

Let $f_0 = \frac{1}{P}$ and $F_{mn} = \frac{w^2}{|P|^4} \operatorname{sinc}\left(\frac{wm}{P}\right) \operatorname{sinc}\left(\frac{wn}{P}\right)$, we have:

$$\tilde{M}(f_x, f_y; x_i, y_i) = \sum_{m,n} F_{mn} \exp\left(-j2\pi f_0 (mx_i + ny_i)\right) \delta(f_x - mf_0, f_y - nf_0). \tag{S29}$$

For circular holes with diameter w and pitch P, $F_{mn} \propto \mathrm{J}_1(\pi w \rho_{mn})$, where ρ_{mn} is the polar coordinate parameter related to mn, $\mathrm{J}_1(\cdot)$ is a first-order Bessel function.

When the structured light passes through the objective lens, the illumination pattern $I_{\mathrm{ILL}}(x, y; x_i, y_i)$ can be represented as the convolution of $M(x, y; x_i, y_i)$ with the excitation PSF $\mathrm{PSF}_{\mathrm{ex}}$:

$$I_{\mathrm{ILL}}(x, y; x_i, y_i) = M(x, y; x_i, y_i) * \mathrm{PSF}_{\mathrm{ex}}(x, y) \tag{S30}$$

$$\begin{aligned}
\tilde{I}_{\mathrm{ILL}}(f_x, f_y; x_i, y_i) &= \tilde{M}(f_x, f_y; x_i, y_i) \cdot OTF_{\mathrm{ex}}(f_x, f_y) \\
&= \sum_{m,n} F_{mn} OTF_{\mathrm{ex}}(mf_0, nf_0) \exp\left(-j2\pi f_0 (mx_i + ny_i)\right) \delta(f_x - mf_0) \delta(f_y - nf_0) \\
&= \sum_{m,n} F_{mn} C_{mn} \exp\left(-j2\pi f_0 (mx_i + ny_i)\right) \delta(f_x - mf_0) \delta(f_y - nf_0)
\end{aligned} \tag{S31}$$

Where OTF is the Fourier transform of PSF. The illumination pattern excites the sample $\mathrm{Obj}(x,y)$, and the emitted fluorescence is finally detected by the camera as the convolution with the detection PSF $PSF_{\det}$ in the spatial domain:

$$I(x,y;x_i,y_i) = I_{\mathrm{ILL}}(x,y;x_i,y_i)\cdot \mathrm{Obj}(x,y) * \mathrm{PSF}_{\det}(x,y) \tag{S32}$$

$$\begin{aligned}\tilde{I}(f_x,f_y;x_i,y_i) &= \tilde{I}_{\mathrm{ILL}}(f_x,f_y;x_i,y_i) * \widetilde{\mathrm{Obj}}(f_x,f_y)\cdot \mathrm{OTF}_{\det}(f_x,f_y) \\ &= \sum_{m,n} F_{mn}C_{mn}\exp\left(-j2\pi f_0(mx_i+ny_i)\right)\widetilde{\mathrm{Obj}}(f_x-mf_0,f_y-nf_0)\mathrm{OTF}_{\det}(f_x,f_y)\end{aligned} \tag{S33}$$

Therefore, the final imaging result can be regarded as the superposition of multiple frequency domain components, which is similar to SIM. According to the Rayleigh diffraction limit, the highest frequency achievable with conventional widefield imaging is:

$$f_{\mathrm{WF}} = \frac{1}{0.61\lambda/\mathrm{NA}}, \tag{S34}$$

where NA is the numerical aperture of the objective lens, and λ is the emission wavelength of the fluorophore. The modulation frequency of traditional SIM can be set up to:

$$f_0 = \frac{1}{0.61\lambda/\mathrm{NA}} = f_{\mathrm{WF}}\,. \tag{S35}$$

Hence the cut-off frequency reaches $f_{\mathrm{WF}} + f_0 = 2f_{\mathrm{WF}}$. Here considering that the diameter of the pinhole is one airy unit (AU), that is, $w = 1.22\lambda/\mathrm{NA}$, the minimum distance between two adjacent pinholes is $P = 1.22\lambda/\mathrm{NA}$ (not selectable), and the maximum frequency of modulation is:

$$f_0 = \frac{1}{1.22\lambda/\mathrm{NA}} = 0.5f_{\mathrm{WF}}\,. \tag{S36}$$

When retaining the first order and ignoring the noise effects, the supported frequency region can be up to $f_{\mathrm{WF}} + f_0 = 1.5f_{\mathrm{WF}}$. Therefore, referring to the method of decoding and shifting frequency domain components in SIM, the resolution can be improved. However, since the decomposition of frequency components is more complicated, the reconstruction method in this study is still based on the spatial domain.

References

1. Ren W, Ge X-C, Li M-L *et al.* Visualization of cristae and mtDNA interactions via STED nanoscopy using a low saturation power probe. *Light Sci Appl* 2024; **13**: 116.
2. Theer P, Mongis C, Knop M. PSFj: know your fluorescence microscope. *Nat Methods* 2014; **11**: 981-2.
3. York AG, Parekh SH, Dalle Nogare D *et al.* Resolution doubling in live, multicellular organisms via multifocal structured illumination microscopy. *Nat Methods* 2012; **9**: 749-54.
4. De Luca GM, Breedijk RM, Brandt RA *et al.* Re-scan confocal microscopy: scanning twice for better resolution. *Biomed Opt Express* 2013; **4**: 2644-56.
5. Roth S, Sheppard CJ, Wicker K *et al.* Optical photon reassignment microscopy (OPRA). *Opt Nanoscopy* 2013; **2**: 1-6.
6. York AG, Chandris P, Nogare DD *et al.* Instant super-resolution imaging in live cells and embryos via analog image processing. *Nat Methods* 2013; **10**: 1122-6.
7. Schulz O, Pieper C, Clever M *et al.* Resolution doubling in fluorescence microscopy with confocal spinning-disk image scanning microscopy. *Proc Natl Acad Sci USA* 2013; **110**: 21000-5.
8. Azuma T, Kei T. Super-resolution spinning-disk confocal microscopy using optical photon reassignment. *Opt Express* 2015; **23**: 15003-11.
9. Castello M, Tortarolo G, Buttafava M *et al.* A robust and versatile platform for image scanning microscopy enabling super-resolution FLIM. *Nat Methods* 2019; **16**: 175-8.
10. Guan M, Wang M, Zhanghao K *et al.* Polarization modulation with optical lock-in detection reveals universal fluorescence anisotropy of subcellular structures in live cells. *Light Sci Appl* 2022; **11**: 4.
11. Ingaramo M, York AG, Hoogendoorn E *et al.* Richardson-Lucy deconvolution as a general tool for combining images with complementary strengths. *Chemphyschem* 2014; **15**: 794-800.
12. Ströhl F, Kaminski CF. A joint Richardson-Lucy deconvolution algorithm for the reconstruction of multifocal structured illumination microscopy data. *Methods Appl Fluoresc* 2015; **3**: 014002.
13. Hansen PC, Nagy JG, O'leary DP. *Deblurring images: matrices, spectra, and filtering*: SIAM, 2006.
14. Chen L, Wang M-Y, Zhang X *et al.* Group-Sparsity-Based Super-Resolution Dipole Orientation Mapping. *IEEE Trans Med Imaging* 2019; **38**: 2687-94.
15. Beck A, Teboulle M. A fast iterative shrinkage-thresholding algorithm for linear inverse problems. *SIAM J Imaging Sci* 2009; **2**: 183-202.
16. Hayashi S, Okada Y. Ultrafast superresolution fluorescence imaging with spinning disk confocal microscope optics. *Mol Biol Cell* 2015; **26**: 1743-51.
